# Improving brain computer interface research through user involvement - The transformative potential of integrating civil society organisations in research projects

**DOI:** 10.1371/journal.pone.0171818

**Published:** 2017-02-16

**Authors:** Bernd Carsten Stahl, Kutoma Wakunuma, Stephen Rainey, Christian Hansen

**Affiliations:** Centre for Computing and Social Responsibility, School of Computer Science and Informatics, De Montfort University, Leicester, United Kingdom; Utrecht University, NETHERLANDS

## Abstract

Research on Brain Computer Interfaces (BCI) often aims to provide solutions for vulnerable populations, such as individuals with diseases, conditions or disabilities that keep them from using traditional interfaces. Such research thereby contributes to the public good. This contribution to the public good corresponds to a broader drive of research and funding policy that focuses on promoting beneficial societal impact. One way of achieving this is to engage with the public. In practical terms this can be done by integrating civil society organisations (CSOs) in research. The open question at the heart of this paper is whether and how such CSO integration can transform the research and contribute to the public good. To answer this question the paper describes five detailed qualitative case studies of research projects including CSOs. The paper finds that transformative impact of CSO integration is possible but by no means assured. It provides recommendations on how transformative impact can be promoted.

## 1 Introduction

Brain computer interfaces (BCI) have been researched for several decades. Progress in neuroscience, computer science, and in the development of supporting technologies has led to the situation where these technologies now have the potential to be widely used. Application areas of BCIs are broad. However, the focus of much research and many applications are medical. BCIs hold the potential to offer support to severely ill or disabled users, including stroke patients, sufferers of cerebral palsy, and locked-in patients. These medical applications demonstrate that BCIs have the potential to contribute to the public good and benefit disadvantaged members of society. It is therefore plausible to assume that involvement of civil society organisations (CSOs) that represent such potential users could strengthen the interests of these users and improve both the quality of BCI research and its outcomes. Using empirical case studies this paper investigates whether and to what degree such involvement of civil society organisations can shape or even transform BCI research. We use the term CSOs in this paper even though it can be confusing or unclear. While some CSOs we interviewed were aware of the term, others were not. The term is often used interchangeably with similar other terms like non-governmental organisations (NGOs), not for profit or non-profit. The European Union (EU) defines it in broad terms as ‘Any legal entity that is non-governmental, non-profit, not representing commercial interests and pursuing a common purpose in the public interest’ (http://ec.europa.eu/research/participants/portal/desktop/en/support/reference_terms.html, accessed 02.12.2016). This is not entirely helpful as it potentially leaves out commercial entities who are interested in pursuing an agenda for the public good. However, as the EU is a major research funder and uses the terms CSO in pushing its agenda for public engagement in research, the authors adopted it for the purposes of this research. Instead of uncritically accepting this definition, we contributed to the clarification of the term. In order to achieve this we asked participants about their understanding of the term. The concept of CSO is revisited on this basis in the conclusion.

The question of the influence of CSOs on BCI research relates to a broader debate of the relationship between science and society. Traditionally the established view was that science and technology research were expert activities that received support and funding from public and private sources in exchange for the production of socially desirable outcomes, such as knowledge and technical products. This traditional view has been contested for several decades, based on the argument that not all consequences of research and technology development are beneficial and desirable. As a result, it has been argued that society needs to play a more significant role in research. There is a long debate concerning the form that this role could take. Recently there has been a strong push by research funders and policy makers, notably the European Union in its Horizon 2020 funding programme, to integrate CSOs in research. The idea behind this integration is that the presence of stakeholder representatives within research projects will ensure that societal needs are met. The consequences are furthermore intended to be not just marginal or incremental but there is hope that they can be transformative for the research itself as well as its outcomes.

Despite a significant policy push for CSOs involvement in research, including BCI research, very little is known about the consequences of such integration in practice. It is neither clear whether such integration achieves its political aims nor whether and in what way it makes a material difference to the quality of research and its outputs. It has, however, been argued that a successful collaboration of various stakeholders in research is most likely in those cases where the subject area allows their interests to combine, i.e. in those areas that visibly affect the public good and that are of societal interest. BCIs are one such area due to their promise of unique benefits to disadvantaged members of society that otherwise cannot be achieved. We therefore chose the research field of BCIs to investigate empirically which influence CSO inclusion can have on research process and outcomes. In particular we wanted to understand whether such involvement can be transformative or whether its consequences are incremental or negligible.

In order to answer these questions, we start the paper by briefly introducing the subject area of BCIs. In addition to the definition of this technology we discuss the concerns that BCIs give rise to and the benefits they promise. Following this we give an overview of the discussion of the relationship of research and society with a particular emphasis on the role that CSOs can take in research, according to the literature. In this section we define indicators of CSO impact on research that allow us to empirically measure the relevance of CSO involvement. Based on these conceptual foundations we then describe five case studies of BCI projects which included CSOs. By undertaking a cross case analysis of the rich qualitative data collected on these cases we use the indicators developed earlier to investigate the degree to which CSO involvement can be seen as transformative. The discussion of the cross-case analysis reveals the factors that promote or inhibit the successful integration of CSOs and their influence on research. The conclusion highlights limitations and further research and highlights recommendations that facilitate the successful inclusion of CSOs in research.

Academic discussions concerning public engagement in general and CSO involvement in particular have a long history. This paper’s unique contribution to knowledge is that it draws on empirical evidence to describe the social reality of CSO involvement in specific research contexts. The paper provides a comprehensive overview of the factors that could be empirically identified as enabling or hindering CSO involvement in five BCI research projects. On this basis the paper contributes to the improvement of BCI research by providing examples of how CSO involvement can transform and improve projects. It provides practical recommendations for various stakeholders on how to improve the situation. It furthermore makes an important contribution to research governance and research policy more broadly by providing a conceptual foundation and empirical evidence concerning the implementation of public engagement by CSO involvement.

## 2 Brain computer interfaces

In order to understand how BCI research projects can be transformed by CSO inclusion it is necessary to understand the technology in question. We therefore provide a brief definition of BCIs and discuss possible concerns they may raise. Such concerns are a key reason for the involvement of CSOs.

### 2.1 Definition

A brain computer interface (BCI) is a technology that allows the user to interact with a computer using only their brain. This is typically done by capturing brain activity using methods such as, but not limited to, electroencephalography (EEG). One can sometimes find the term brain neuronal computer interfaces (BNCI) in the literature. BNCIs expand the definition of brain computer interfaces to include interfaces that use other physiological activity. BNCIs are sometimes referred to as hybrid or multimodal BCIs [1]. In this paper we will be referring to cases where researchers used this term instead of BCI. However, these two definitions are closely related and the differences are not material to the argument of this paper. We therefore use the more broadly used term BCI but include BNCIs in them.

A BCI consists of a data acquisition part, which captures and digitizes brain signals for a processing system. This signal processing system then provides instructions to the device or interface being controlled by the user [2]. The classification of different BCIs can be based on various perspectives, such as mode of capture, or usage of lubrication [3,4].

Invasive BCIs are interfaces that monitor brain activity from inside the skull of the user. These BCIs have a high precision as they are able to monitor brain activity directly, and can be fitted in a way so they are able to capture individual neurons' activity. These devices are more complicated to use than non-invasive BCI, as they require surgery and require the user to be in collaboration with medical staff to ensure that no health issues arise from the implant. A non-invasive BCI, on the other hand, is an interface which monitors the brain activity without penetration of the skull of the user. These devices come in a varying degree of precision, which often depends on the number of electrodes employed and whether lubrication is used. A non-invasive BCI is easier to use than an invasive BCI and can often be setup either by the user alone, or with the help of another person. This could be a caretaker or a family member, and does not require medical training [5,6]. While invasive BCIs have potential applications in medical contexts, they are unlikely to be used more broadly. All of the case studies investigated in this paper made use of non-invasive BCIs.

### 2.2 Potential benefits and concerns

BCIs have the potential to significantly improve the lives of some users, offering them abilities to act and communicate that are otherwise not available to them. However, they can also raise concerns which need to be understood, in order to gain an understanding of the role the CSOs can have in BCI research. In the following subsections we provide a brief overview of concerns that have been raised in the literature. We do not pass judgement whether these are realistic. The purpose of the section is to demonstrate that BCIs raise questions that may warrant broader societal outreach during BCI research and development.

#### 2.2.1 Privacy

Privacy is one of the obvious ethical concerns when dealing with any information technology. Privacy concerns have been scrutinised with extensively within the literature [6–10]. The new aspect introduced by the nature of brain computer interfaces arises from the kind of data produced by these devices. Users are not only able to collect data produced by conscious actions, but all the data produced by the user’s brain. This introduces new concerns where the data produced can provide information that users does not even know about themselves [11–13]. This is a unique challenge for BCIs and privacy concerns. Some scholars are trying to create frameworks for secure BCI that allows for privacy [14,15]. Whether these frameworks and developments will succeed is still to be seen.

#### 2.2.2 Discrimination

There are two major concerns regarding discrimination and BCI usage. The first concern is discrimination against BCI users. This concern mainly focuses on discrimination against BCI users which parallel the concerns of wheelchair users, hearing aid users and the like. This concern is something that research settings have raised as a major concern for BCI users, which has suggested that effort should be put into creating BCI that can be worn without making it obvious that the user is wearing one. [16,17] The second concern is discrimination based on data created by BCIs. This sort of discrimination has been debated in terms of the usage of BCI as lie detectors, however is also part of the privacy debate as one of the reasons for wanting privacy is to avoid misuse and discrimination based on BCI data [13,18,19].

#### 2.2.3 Digital divide

The ethical question of digital divides with regards to BCIs is especially relevant concerning the topic of enhancement [18,20]. What is interesting with regards to BCI devices is whether there will be a similar ethical concern, and what could be done, if BCI were to become a common work place tool comparable, say, to email [21]. At the moment BCIs can be bought for prices between 71€ and 359€ for end user versions, which might seem like a low entry point [22,23]. And this concern is in some sense minimized by the technology moving to commercial use as BCI providers will have an interest in as many people as possible buying their devices. However this shifts the problem for digital divide concerns from obtaining the device to the concern of whether people are able to use it. Current research is showing that some individuals are unable to use BCI devices [24]. This creates the question of how to close this gap between people who are able to use the devices and those who are not [25].

#### 2.2.4 Enhancement

There has been general debate about enhancement, including brain enhancement in various ways [20,26]. Much current research focuses on the risk of enhancement in general such as long-term effects and what could be considered augmentation, beyond rehabilitation [27].

#### 2.2.5 Autonomy

Autonomy is another ethical concern, mainly discussed in the literature in terms of the implications of lie detectors (and privacy), or on the topic of justice [28–31]. The ethical concern is usually directed towards the idea of identity and individuals’ autonomy, and how these terms change if (or when) neuroscience shows that all our actions can be scientifically determined. This raises questions for entire justice systems, and whether we are responsible for our actions if everything is determined by our biology and brain signals. The other less direct problem with autonomy is when BCI becomes a tool for employers to determine whether someone is suitable for a job [32]. This could reduce our autonomy as some jobs would then become unavailable based on inferences made from our brain signals. In a similar way, researchers have argued about neuromarketing as one of the fields which could reduce the autonomy of individuals. [33] In research settings handling locked-in patients there is also a concern for keeping the individual’s autonomy as there are attendant issues about getting informed consent [34].

### 2.3 A BCI application scenario

These remarks about ethical and social concerns of BCIs do not claim completeness or comprehensive coverage. In order to understand how these technologies may be used and which questions that can raise, we now present a brief application scenario. This scenario is based on some of the shared characteristics of the five case studies which we develop in more detail below. Its purpose is to show the reader why and research and development of BCIs go beyond purely technical questions. This wider interest is the reason for involving CSOs and the basis of the research question behind the paper.

BCIs can be seen as alternative input devices comparable to a computer mouse or keyboard. In this sense they can be used for a virtual infinity of purposes and applications. However, at present their complexity and expense means that they are most likely to be used in cases where other input and interface devices are not available. Our application scenario therefore focuses on potential users who are not able to use other interface devices. This is the reason why BCI research focuses on ill or disabled individuals. In our application scenario we assume that this target group are stroke patients who have lost the use of their upper limbs, which is one of the groups we encountered in the case studies. The scenario can then be developed to outline the intended benefits for these users. By being able to access computers via a BCI, such stroke patients can regain capabilities such as interacting with friends and family on social media or using medical application to track the progress of their recovery. BCIs thus offer the possibility of increasing these individuals’ freedom and autonomy [35], which is without doubt a valuable goal worthy of any research project.

Despite such advantages the concerns outlined above need to be taken into account. BCIs require data that users may find sensitive and are unwilling to share. Users may be unwilling to use such devices because they are conspicuous and can single them out as disabled beyond what they already encounter, e.g. by using a wheelchair. Users may reject this technology as it can serve as a continuous reminder of their condition. Maybe even worse, they may pin their hopes on these new technologies only to then find out that they don’t work for them, as can be the case for some users [36,37]. Alternatively, users may find out that the BCI works for them, but then they find themselves in a situation where they become dependent on the device or where support cannot be delivered beyond the end of the project. In addition to such individual questions there are larger questions concerning who should benefit from BCIs, and who ought to draw up decision principles concerning under which circumstances the device should be withdrawn.

The purpose of this brief application scenario is to show that many of the questions that are relevant to BCI research and development are not exclusively technical questions. They are questions that researchers and technicians cannot answer by themselves. They also in many cases go beyond more traditional usability questions that can be explored through traditional usability or HCI methods. They are questions that require the input of those stakeholders that are affected and that understand not only the immediate context of use but also the broader environment. These are questions which may benefit from insights by non-research experts who represent civil society. We therefore now look at the discourse concerning the role of civil society organisations in research.

## 3 CSOs and public engagement in research

Having defined the technology of BCIs and the types of concerns they may raise, we can now look at the role that CSOs can play in BCI research. In a first step we briefly sketch the background of public engagement and the role of civil society in research in general. This provides the basis for the definition of CSOs and a discussion of their possible roles in research. On this basis we suggest criteria that could be used to assess whether CSO integration into research has a transformative impact.

### 3.1 Public engagement: The role of society in research

The relationship between science, research and society has changed significantly over time. Originally the pursuit of the ‘gentleman scholar’, science and research have been transformed into an integral part of the socio-economic fabric of society. Vannevar Bush’s [38] vision of the role of research as the driver of social innovation dominated the post-world war II era. It was based on the idea that publicly funded research is free to pursue its interest but in return provides society with desired innovation and the trained scientific workers. This post-war division of labour did not last. Disenchantment with the consequences of research and technology development led to different views.

Critical scholars pointed out that technologies, including assistive technologies aren't politically, socially or cognitively neutral [39]. Politically, they manifest agendas that steer funding toward some goals rather than others. Socially, they can change the ways in which individuals and groups interact, via communication devices for example.

The recognition that science and technology are politically charged led to a call for stronger links with society at large. This was particularly strong in those cases where the research was publicly funded or had large-scale consequences, such as in the case of nuclear technologies or genetically modified organisms. While the importance of including broader publics in science has gained acceptance, there remain various interpretations of the role that public participation should have and the form it should take. Initially dominant was the “deficit model” which assumes that the problem lies with a lack of understanding of science by the public [40]. This deficit can then be overcome by communicating effectively; a suggestion captured in Dudo and Beasley’s article on scientists’ prioritization of communication objectives for public engagement [41].

It has been recognised for some time now, however, that the deficit model is insufficient and that public concerns about research and technology development can be well justified and informed. Public engagement can serve to improve acceptance [42] but can have further goals. It can be used to make use of local expertise of the affected publics which can improve the research knowledge base by overcoming the limitations of experts [43], injecting creativity into research [44] or improving quality control [45]. These activities suggest a very different relationship between research and technology development and society at large, which is often referred to as mode 2 of engagement, which is more egalitarian and has a broader social relevance than the traditional mode 1, which is investigator-driven and discipline-led [46]. Another key difference between mode 1 and 2 is in terms of accountability—Mode 1 research is accountable in terms of the discipline whereas mode 2 can be held accountable by a potentially diverse group of parties, across a variety of disciplines, areas of study and other fields of interest.

Mode 2 engagement has gained significant traction among research funders and policymakers, partly because of its democratic potential to transform research into an activity that explicitly addresses social issues. On a European level, engagement furthermore promises to implement Article 11 (1) and (2) of the Treaty on the European Union that hold that

“1. The institutions shall, by appropriate means, give citizens and representative associations the opportunity to make known and publicly exchange their views in all areas of Union action. 2. The institutions shall maintain an open, transparent and regular dialogue with representative associations and civil society.”

(see: http://eur-lex.europa.eu/legal-content/EN/TXT/HTML/?uri=CELEX:12012M/TXT&from=EN, accessed 09.10.2015)

Despite this strong policy rationale for deep (mode 2) engagement with society in all public activities, including research, it is not clear how such engagement is to be put in practice. As Nowotny and her co-authors [47] put it: "…politicians and civil servants [are] struggling to create better mechanisms to link science with innovation”. Existing incentive structures within the scientific system do not always support societal engagement. Further problems and downsides of public engagement are manifold and described in the literature; they include additional pressures on time and budget [40], a lack of willingness by public authorities to engage [48], cultural differences between researchers and the public, as for example expressed by the scientific focus on publishing [49], problematic social dynamics [50] including the possibility of subversion of engagement for particular purposes [51]. Where engagement is meant to create legitimacy of research and its findings [45,52], this raises questions about the representativeness of the participants and the relationship between local engagement and representative democracy.

Despite these many questions and pitfalls of it is worth pointing out that the discourse and practice of public engagement have matured considerably [53]. Drawing on long histories of engaging with various publics in the design and evaluation of research and technology, e.g. through participatory design [54], participatory systems development [55,56], participatory technology assessment [57], participatory action research [58,59] as well as numerous participatory political activities, a large number of formats of public engagement are now available. The European project Engage 2020 (http://engage2020.eu/, accessed 13.06.2016), for example lists 57 different methods and provides detailed descriptions of their applications and strengths and weaknesses. These methods are general public engagement methods, however, and do not necessarily give guidance on how to involve the public in research. This is where the idea of integrating CSOs as representatives of civil society in research enters the debate.

### 3.2 CSOs in research

The term CSO is ambiguous and often used interchangeably with such terms as Non-governmental Organisation (NGO), Voluntary Sector or Third Sector to denote organisations working towards the public good outside the state or business sectors on a non-profit basis for purposes of giving a voice to the voiceless and to keep the State in-check [60][61]. CSOs are looked upon favourably by both policymakers [62] and individuals [63] as contributors to policymaking. They are seen as alliance-brokers between public and policymakers [64,65]. This ability to participate in agenda-setting at a policy level is reflected in the ambition that CSOs’ expertise be included at research project-level agenda-setting [40,66]. Traditionally, CSO partners fulfil a dissemination role [67] which can be attributed to their acknowledged excellence in communicating science to the public and societal groups [64,68]. Another aspect of this communicative excellence is the sectoral knowledge and oversight CSOs can provide in research [63,69].

CSOs can function as co-producers of knowledge [70] which helps them to communicate scientific knowledge in a language that the sectors they represent understand. Rather than view knowledge as the preserve of an elite few scientists, CSOs make it accessible to ordinary citizens. Doyle and Patel [71] show how CSOs have played a role in global health governance where they have been seen as key players and have thus been consulted by international organisations like the World Health Organisation (WHO) in finding solutions to ailments like malaria, HIV/AIDS as well as Tuberculosis. CSOs or groups or individuals within civil society such as patients can be seen as strategic partners who have the capacity to build a bridge of understanding between the public and policy makers [72] [73]on various activities, be it research or holding governments to account. Most of these abilities are rooted in the trust that CSOs can engender for the aims and practices of research projects [74] and the perceived legitimacy of research project outputs [75].

### 3.3 CSOs and BCI research

Despite these general advantages of CSO involvement in research, their involvement in BCI research is still in its infancy. The literature suggests that, although CSO involvement has been apparent in trying to achieve some public good in the spheres of health, human rights and environment, typically their involvement has had little to do with BCI research. This is perhaps due to the idea that the public as seen through scientists’ eyes view emerging technologies as too risky [76]. Furthermore, the literature also shows that there has been little emphasis on calls for public engagement in research through CSO participation until now [77] [78].

However, this appears to be changing, with the desire to have a more democratic approach and inclusion of public opinion in the design and execution of research, especially where it is intended for the public good and its end-results are expected to make a direct and/or immediate difference to people’s quality of life [73] [79] [80]. BCI is one such field, where the focus is on the technological and scientific developments to directly improve the human-computer interaction, e.g. with an aim to facilitate and enhance the communication of disabled people with and within their environment.

As a result there has now been a number of BCI research projects that have included various types of CSOs (see case studies, below). Inclusion by itself, however, does not guarantee that the change of consortium composition and research structure improves or changes the research. We therefore now discuss how one can assess whether CSO inclusion has made a difference to a research project.

### 3.4 Indicators of transformative impact

Transformative impacts are those that affect the trajectory of research and innovation to a high degree. The linear model of innovation [81] portrays the progress from research to innovation as a straight line [82]. Research is followed by technology development, and then innovative new products are built on the back of the new technologies. The end point and marker of progress, of all of this is economic success. Research is *worth it* when it results in marketable technologies.

While the linear model may be useful in some circumstances, it has been shown to be empirically deficient [83,84]. It is a model with a purpose that presupposes a great deal about what constitutes a ‘good’ outcome (i.e. innovation posits research and development leading to marketable technologies as a good thing) [84,85]. Real-life innovation is more complex and messy. This kind of complexity is followed up on in Garud and Rappa [86] as they describe the technological evolution of the cochlear implant, offering a critical model to evaluate it. Their work suggests that in the midst of beliefs held by researchers, thoughts concerning the functionality of the technological artefact being developed, and the ways in which the researchers assess their own work are embedded implicit worldviews that create pathways for development.

In the development of technologies, a certain worldview is imported to the technology and around this a shared vision of a world improved by the technology is constructed. Often missing is a point of ‘negotiation’ with other worldviews from whom can be gained more information on the world as it is and on its possible futures. By bringing in novel voices in this negotiation, research can be transformed. CSOs as organisations that follow different logics and have different purposes from research-oriented organisations can play a role of changing the negotiation and joint construction of a view of a technology in a broader context.

Essentially, the elements relevant to the context of assessing transformative impact are “beliefs, artifacts [sic] and evaluation routines” [86] across a spectrum of relevant parties. Central in this scheme of things, when considering transformative research, are the beliefs of researchers. The key beliefs concern:

- Possibilities and expectations
  - What can be done and what is desirable?
- The technologies themselves
  - What is their usability, their contribution to users’ lives?
- The adequacy of the assessments of the technologies in light of possibilities and expectations
  - What methods or routines are relied upon to connect researcher beliefs to user realities?

Transformative impacts will be those that affect the beliefs reflected in these three points. In terms of the development of technology, these are the facets that fundamentally shape the trajectory from idea, to lab, to market, and so to the user. The sectoral, legitimising and communicative potential of CSOs may provide just the input needed to negotiate the trajectory of technology development here.

The beliefs held by researchers concerning what can be done, and what is desirable to do, will be at least partly determined by the worldview they hold. The mere inclusion of perspectives other than researchers’ may not lead to transformative impact. CSOs may sometimes be included as mere audiences for research, rather than full participants [87]. The types of inclusion as well as the role of the CSO are also relevant features [53,88]. Transformativity comes from the connection with other views and the testing of prior (perhaps tacit) beliefs. For this connection to be effective, the locus of control within the research must be carefully constructed. The locus of control can also, therefore, be used as an indicator of the possibility for transformational impact. This can be evaluated through an examination of the roles played in research by the various parties (e.g. funder, research agenda setter, PI, dissemination partner, advisory board member). The role influences power, and so can stand as a proxy for the locus of control. From these considerations, a general scheme for considering transformative impact in a research trajectory suggests itself as below.

**Fig 1:**
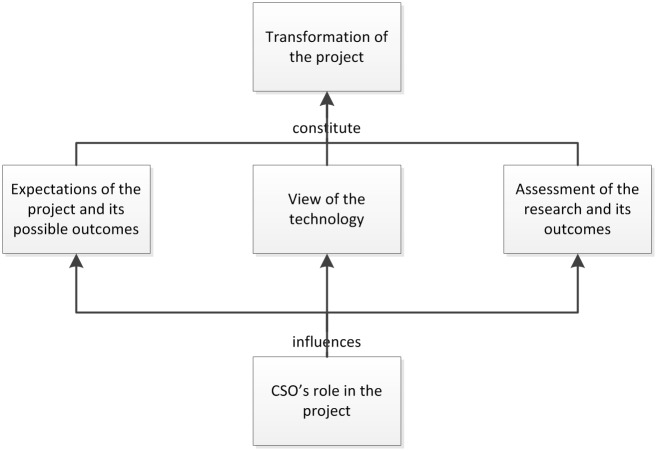
Graphical representation of components of transformativity.

This schema of transformativity can be graphically represented as follows in Fig 1:

Having thus established a set of possible indicators that could show whether CSO inclusion has a transformative influence on a project, we can now proceed to explore these using five case studies of research projects on BCIs that included CSOs. For the sake of simplicity of description, the case studies contain a brief narrative part and then compress the findings that allow a determination of the level of transformative in a table representing the components just described. A sample of such a table without any content can be seen in Table 1:

**Table 1:** General scheme for assessing transformativity.

	Researcher	CSO	Other (companies or funder)
Expectations			
Technology			
Assessment			
Role			

This general schema was used to interpret our data after the data collection was finished. Its specific purpose was to guide one step of the data analysis to allow us to determine whether there was evidence of transformativity in the project. We will now use this schema to investigate whether CSO integration into BCI projects shows empirical evidence of a transformative impact.

## 4 Comparative case analysis

In this section we compare five BCI research projects to explore whether CSOs had a transformative influence on the research, and whether it is possible to identify factors that contribute to such transformative impact or impede it. Before we discuss the individual cases we outline the methodology used to collect and analyse the data. This is followed by an introduction of each of the five cases which leads to a comparative discussion.

### 4.1 Methodology

This research explored novel relationships where currently no clear views or hypotheses exist. It therefore employed an interpretive approach [89,90] using qualitative data [91] to gain insights into the perceptions of the individuals involved. The transformative impact of CSO involvement is most easily observed on the level of the individual project. We therefore decided to follow an interpretive case study approach [92] using the individual research project as the unit of analysis.

The starting point of our methodology development was that, despite a rich discourse on public engagement in research, there was very little empirical work on how exactly CSOs are involved in research and which mechanisms and interactions can play out in actual research projects. In order to understand the internal logics of research projects, we therefore employed a research strategy inspired by Grounded Theory (GT) [93,94]. This means that we refrained from forming pre-set hypotheses or data analysis structures. Instead, we aimed to keep an open mind and develop the data collection and analysis according to our developing insights. The research team are aware of the strong influence of the researcher on the findings and we would classify our approach to GT as one based on a constructivist understanding of research [95]. As we used a shared set of top level codes that were refined during the research, we realise that we did not follow the principles of GT. This is why we point out that the research was “inspired” by GT, i.e. used many of the principles, but cannot pass as an implementation of GT. The study was nevertheless interpretive in that it attempted to “understand phenomena by observing and describing the meanings that people assign to them” [96]. We incorporated the principles of phenomenology and hermeneutics, two basic epistemological philosophies that inform interpretive research [97–99].

It is important to point out that the research was undertaken in the context of a European project (Civil Society Organisations in Designing Research Governance, CONSIDER, www.consider-project.eu) and that the data and interpretation presented in this paper cover only a subset of the work undertaken in the CONSIDER project. The research design and implementation was a distributed effort undertaken by the partners of this project. As not all aspects of the CONSIDER project are relevant to this paper, we only report those components that are required to understand the overall narrative of this paper.

#### 4.1.1 Data collection

Following the development of the research strategy, we developed appropriate consent procedures and received ethics approval for the study from the various institutional research ethics committees involved. The ethics approval was led by the French partner, the University of Lille, which also registered the study with the French data protection authority, the CNIL. All data was processed in a de-identified format, i.e. all names and identifying properties were removed. However, due to the specificity of the case, identification of the individuals would still have been possible. It was therefore decided that the full case study descriptions would have to remain confidential (see S5 Appendix for the possibility of data access).

Cases were selected on the basis of an earlier survey of all research projects that had been funded up to that point under the European Union’s 7^th^ Framework Programme (FP7). This earlier survey had allowed the identification of around 400 projects that included CSOs. From these we selected a sample including EU and non-EU funded projects, all of which were conducted in European countries, however. Overall, we conducted 32 case studies across a large number of fields and disciplines. The detailed case studies were selected from the population of 400 on the basis of their distribution along two variables: the role of the CSO in leading the project and the intensity of interaction between the partners. By mapping these two variables, which were assessed on the basis of the initial survey, we aimed to select a range of projects that would represent significantly different practices in CSO integration. More information on the initial survey, the selection process and subsequent empirical investigation is available in [100]. In this paper we describe a subset of these 32 cases, namely cases of research projects that had BCIs as their research focus. The cases thus had the technology under investigation in common but differed with regards to intended use and application as well as organisation and location of project partners.

For each case study it was decided to collect the following data:

- Minimum requirements for each case:
  - Three interviews with
    - Project Coordinator / PI
    - Scientist
    - CSO
    - at least 1–2 of these interviews should be done face to face
  - other documents, if available
    - website
    - project reports
    - proposal
    - project presentation
    - call document
    - organisational structure / chart
- Desirable data
  - Deliverables (where relevant to CSO participation
  - Observation of project meeting
  - publication
  - promotional material
  - newsletters
  - flyers
  - brochure
  - other interviews
    - former partners
    - partners who declined
    - observe event
    - sponsors / POs
    - industry partners where available

The key data that promised to provide deeper insights into the role of CSOs in research were the interviews. All interviews were conducted by experienced researchers, following a central training session to ensure comparability of approach. The interviews conducted for the BCI cases described in this paper were undertaken by the project coordinator and a senior research fellow.

The interview questions (see S3 Appendix) were developed with a view to gaining insights into key factors affecting CSO participation. They were refined during a pilot case study and further reviewed during the course of the data collection. They focused on the following aspects:

- Description of the overall project
- Meaning of the CSO participation for the overall project
- Definition of CSO
- Selection criteria for project partners
- Roles of the partners
- Conflicts
- Interviewee-related questions
- Follow-on

The incentive offered to respondents was to help them reflect on their project and thereby potentially improve their research. No financial or other incentives were offered. All interviews were fully transcribed prior to analysis. Respondents were offered the opportunity to check the transcripts for correctness. However, this offer was rarely taken up. All interviews undertaken for the five case studies described in this paper were undertaken in English and therefore did not require translation. Data analysis was undertaken by the researchers who collected the data. The case study reports were compiled by the researchers who collected and analysed the data. All case studies were then part of a peer review system by project partners to ensure quality and consistency of the case study documents.

The following Table 2 contains the meta-data concerning the data collection for the five cases discussed in this paper.

**Table 2:** Data on case study projects.

	Case 1	Case 2	Case 3	Case 4	Case 5
Funding source	National research council- Significant co-funding from CSOs (~€400k) and in-kind contribution from industry	National research funding council	EU FP7	EU FP7	EU FP7
Budget	n/a	Overall over €20m with a funding contribution of €10m	€3M with funding contribution of €2.3M	€ 3.2M with funding contribution of € 2.5M	€ 4M 186 248 with funding contribution of € 3 M
Data collected	interviews (Proposal writer-academic researcher Project manager CSO President PI-senior researcher Additional interview with proposal writer Project website Project brochure	interviews (Principal Investigator Sub-project coordinator Senior researcher Industry partner CSO partner) Project final report Project website Journal publication on ethics and the research Project brochure	interviews with 5 respondents (CSO number 1 employee Project coordinator Researcher and WP leader 2 CSO number 2 employees) 9 publicly available deliverables 4 dissemination documents (booklet, publishable summary, poster) project press releases 19 publications 3 videos Project website, consisting of 19 pdf documents Funding call ICT 2009–4	3 interviews (Project Coordinator CSO Researcher Project call Media coverage Deliverables Publications Promotional material	4 interviews (CSO Technical Industry Representative Academic Scientist Project Coordinator Project website
Timing of data collection	Between 23.01.2013 and 08.08.2013	Between 20.12.2012 and 01.03.2013	Between 18.03.2013 and 16.05.2013	Between 22.04.2013 and 20.09.2013	Between 01.07.2013 and 10.04.2014

#### 4.1.2 Data analysis and reporting

In order to facilitate a consistent analysis of the data, the researchers used the qualitative data analysis software NVivo server, version 10. This allowed distributed analysis of the data by the various partners of the CONSIDER project, using one centralised database and identical coding schemes.

The research team had a weekly meeting to discuss progress of data collection and analysis. This meeting was minuted in a case analysis handbook which contained the agreed principles of data collection and analysis as well as the outcomes of discussion. Of particular importance here was the discussion of and agreement on coding nodes. In a normal GT data analysis the research follows several coding steps (e.g. open coding, axial coding, and selective coding) which start with a very fine-grained analysis of the text and builds up to a theoretical representation of the phenomenon. In our project, in order to facilitate collaboration and reduce complexity, it was decided to start the coding with the following set of top level codes

- Collective experiences
- CSO
- Expectations
- Influence factors
- Project
- Recommendations
- Relationship dynamics
- Respondent
- Rhetoric

Where required, researchers could add further sub-nodes, but any new node had to be discussed and agreed by the project partners. This served the purpose of limiting the number of nodes and thereby the complexity of data analysis. The full coding tree is provided in S2 Appendix.

In order to allow for a comparison across the different cases, we developed a template to be used to report each individual case. This template combined descriptive data on the case, the perceptions of the various groups and stakeholders as well as analytical insights won from the individual case. The headings of the template are provided in S4 Appendix.

As a result, each case led to the construction of a case study report. The five reports developed for the BCI cases form the basis of the case analysis provided in the following section.

### 4.2 Case descriptions

To understand whether CSOs have the capacity to be transformative in BCI research, this section will analyse five case studies of BCI research including CSOs. The aim of the analysis is to show whether any of the cases were transformative and in what way. The cases under consideration are outlined below.

1. Case 1 (DMU C): ICT Support for Disabled Children
2. Case 2 (DMU B): Computers and the Brain (large national project)
3. Case 3 (DMU D): Brain Computer Interfaces for severely disabled users
4. Case 4 (DMU E): Affective Computing for Augmented Communication
5. Case 5 (DMU J): BCI for Home Monitoring

The description of the five cases in the context of an academic paper means that the depth of description is limited and the understanding of the cases remains relatively superficial. However, we provide an overview of each case that explains the context of use of technology, which is important to understand why a CSO might be interested in contributing to the research. Wherever possible we also provide quotes from our respondents describing key aspects and insights. Citations are indented and italicised, in order to allow the reader to easily identify them. In addition, within the five case studies, we also discuss the concept of CSOs and how it was understood by participants from the case studies. This is important because as indicated in the introduction, the term CSO is not always familiar to everyone and is sometimes understood through use of different other terminologies such as NGOs, not for profit or non-profit. It was important to ensure that there was an understanding between the authors of the paper and the research participants in terms of what was being researched and why.

#### 4.2.1 Case 1: ICT support for disabled children

This case is about a research project focussed on BCI-controlled orthoses, for the correction of limb disorders through supporting the upper limbs of children affected with muscular dystrophy. As the Coordinator of the project revealed during the interview:

*The project is about developing an arm-assisting device for people with muscular dystrophy, like they are not able to move their arms anymore. So this is someway that they need to continue their daily things even when they become a bit older. Duchenne is progressive, so in the beginning people are able to move their arms but at certain moments they are not able to do it anymore. The goal of the project is to develop a wearable arm support of a fleece or a jacket that you can wear which provides gentle support so there are no extra extensive exoskeletons or robotic devices or huge mechanicals*.

The initial idea of the project was born from a parent whose child was affected by the debilitating Duchenne muscular dystrophy. In their quest to find help for the child, the parent came across some technology on the internet that they thought could be applicable in easing their child’s health problem. The parent was a member of a CSO representing parents of sufferers of the disease. The CSO had significant research experience which it gained through being involved in medical research projects and rallied other CSOs representing patient organisations to set up a foundation to secure funding and develop a research proposal to customise the technology seen on the internet for patients with children with similar problems. The CSO played a significant role in both the conception and development of the project. This was in addition to providing significant funding for the project which, in turn, allowed the project to successfully bid for national research funding. Although the CSO had little formal involvement in the structure of the project, it had a dominant role in terms of ensuring that the project was of high quality in its desired outcomes which was to have a technology product that would support disabled children. The relevant aspects concerning transformativity are summarised in the following Table 3:

**Table 3:** Summary of case 1.

	Researcher	CSO	Funder
**Expectations**	Academic research outcomes, specifically 4 PhDs in the area under research	Customised BCI technology for Duchenne sufferers	Applied and market oriented research outcomes
**Technology**	Purely focussed research on the subject under investigation in order to have some publications on the possibilities of the technology	Technology intended to relieve the suffering of Duchenne sufferers	Technology to have practical applications to the outside world Technology to be commercially viable
**Assessment**	End-user involvement	End-user feedback	N/A
**Role**	Research	Co-funder Project initiators Creation of organisation for proposal writing Proposal writing Research agenda setting	National research funder

The CSOs’ role in case 1 can be viewed as transformative for a number of reasons which go back to the initiation of the project. The CSOs played a distinct role in the research design and setting the research agenda of the project. In addition, the CSOs took the lead in the development of the project. Their contribution was evident from the conception and inception of the project. Specifically, the CSOs were instrumental in initiating the project through setting up a foundation and then co-funding the project. The fact that the CSOs were able to co-fund the project also meant that it made it easy for the project to bid and subsequently successfully secure national research funding. In addition to co-funding the project, the CSOs shaped and wrote the research proposal that set the agenda for the type of BCI technology they wanted realised for their patients. Although the CSO had a very limited formal role in the organisational structure of the project, the CSO had a dominant role in the development and execution of project tasks and placed high importance on the quality of the project outcomes. The CSOs dominant role can be gleaned from the following interview extract with a CSO representative who incidentally is the parent of the child suffering from Duchenne who was instrumental in formulating the research project:

***CSO Interviewee*:** *… what I also expect is to have something by the end of the year so you know er that is what I said, that is what I feel we should put pressure on there*.

***Interviewer*:** *OK*.

***CSO Interviewee*:** *On the projects yeah and on the other hand you can’t make a prototype if the ideas are not there you know. It’s not that we have err, err, unrealistic expectations, that’s something else, but that is the side where we really feel you know you thought to become a hands on thing so that is, that is why we hope that they will have a first prototype soon*.

***Interviewer*:** *Right so what’s the sort of outcome that you would hope for*?

***CSO Interviewee*:** *I think err at the moment you would have a prototype which can be tested that will give you a lot of insight, you know. If you speak about input from patients it is hard to discuss a theory for patients you know, the patients themselves, as soon as they wear something, or try something they can say well this is not convenient, maybe that could be, I would love to do this. Then you have also more hands on method to discuss a product with the patients. So one it is- prototype is important also to evaluate with patients err better evaluate the ideas running at this moment, they will be important. Second as soon as there’s a prototype it is also easier like now we are discussing funding by a big American organisation, as soon as you have the first prototype it is also err more visible what you are raising funds for, how realistic it is that it is going to be to help the boys and third a prototype is the first step to a product, right? So we want a product so these are three reasons I think it could be helpful and they are learning to make different types, you know, but the first phase will be that it will be something anti-gravity so that means that you aren’t really balanced erm that is the first prototype we expect and later they will kind of put little engines to do more and only anti- gravity and so on, you know we don’t expect versions to be, prototypes there within a year. But it would be nice to have err something more touchable, tangible- how do you call it- to go for yeah*.

On the CSO and patient side, there is a clear expectation that the technological outcome ought to be practical and not necessarily just an academic outcome which could be more theoretical and therefore unusable for the patients. It is for this reason that the CSOs were keen to have a dominant presence in the execution of tasks and the quality of the project outcomes. For them practicality meant more in terms of ensuring that patients would be able to find the technology useful for their day to day lives.

In terms of understanding the concept of CSO, the respondents in this case generally had a detailed understanding of it as seen in the following interview quotes:

***Interviewee 1(CSO)*:** *As far as I understood [CSO] it’s an organisation which is not governmentally funded, is not a company that comes from the society and that gathers money to find projects*.

***Interviewee 2 (Scientific rep)*:** *I think that’s an organisation of people with a common purpose which are not provided by Government or commercial organisations*.

Perhaps the very nature of how the project was conceived and funded (by CSOs) meant that there was a clear understanding of the term CSOs and what their remit was.

#### 4.2.2 Case 2: Computers and the brain

The project underlying case 2 was a nationally funded research project that explored the way in which technologies can be used to help sufferers of neurological disorders. It was a large project with more than 20 partners including universities, hospitals, large and small companies and patient organisations. The overall funding contribution by the national research council was over €10m with a project budget of over €20m.

The project was a scientific research project but it was explicit in stating that its intended impact was scientific, economic and social. The project report explicitly states that the “three core aims of the […] programme were scientific excellence, economic valorisation and societal impact.” The intended impact was aimed at disabled users who could use the technology in order to improve their interaction with their environment. However, the project also aimed at other application areas such as computer gaming. The size of the project allowed it to aim at making a contribution to a national infrastructure in the research area and contribute to world-wide research.

The project aimed at commercially viable products, but the actual marketization of these projects was to be left to stages after the end of the research project itself. The project website claimed that it aimed to facilitate products for both disabled and healthy users. This could be partly achieved by establishing strong connections between industry and researchers. The key aspects of this project are collected in Table 4:

**Table 4:** Summary of case 2.

	Researcher	CSO	Funder / Industry
**Expectations**	The development of neurological stimulation methods in addition to assisting in the development of software to predict current flow in the brain. To undertake more scientific theoretically based research about brain functions. To undertake research that would be attractive to industry in order that it can be packaged as a product For the patient organisations to assist in user tests and application in the specific patient ailment. To have more CSO involvement as a way of winning scarce funding. To realise more informed societal input in research.	Manifest outcomes, partly because of the high importance of “valorisation” in the project. To make an immediate difference to the quality of life of their constituents. Specific funds to be allocated to solving specific problems	Impact of research on society Reduced animal testing Involvement of patients Creation of innovative products.
**Technology**	Technology as a means of proving scientific hypotheses. In the second instance, technology as a product to be used by patients	Technology as practical help for the patients the CSOs represent; this was not realised.	Technical products on the market (not realised) Social impact of research
**Assessment**	Relied on scientific evaluation of project through project review; assessment of findings through scientific peer review.	Practical testing of technologies with users	Project review; valorisation panel looking at broader societal impact
**Role**	Leader, organiser and most active partner.	Link to end users; Required CSO inclusion according to funding call No official role, no deliverables	The consortium included companies but this did not lead to a commercial product by the end of the project life cycle.

The CSOs were included in the project as representatives of end users. In this function they were asked to explain the needs of end users in the beginning. They then had the opportunity to give feedback on project progress during the six-monthly meetings. The project aimed to provide practical outcomes, in line with the requirements of the funder. This was linked to the idea of valorisation. CSOs were involved in valorisation activities. They were therefore included in application development and end user testing. The following interview extract gives a more clear indication of why CSOs were involved in such a project:

***Interviewer*:** *One of the things we are interested in is what effects are expected from the CSOs. Do you know why CSOs were involved, whether there was some sort of deliberative process where the consortium or investigators discussed what they want from CSOs*?

***Interviewee*:** *So what I know from it is that in the Netherlands we have gas in the earth for fuel. Dutch government makes money with it and at some point they had some money to spend. Yeah, it is a very crazy story! Ummm, so they decided that they would want to have research, like applied research, forcing researchers more towards an applied field. So…. smart mix of universities, companies that would like to participate. So there was an incentive from the government to involve patient organisations so that the research would be more applied, also that the patients would give directions for the research to go and talk to them. So I think the main incentive was, you know to be honest the funding was organised, it was demand from the government to include CSOs, to force I think to force researchers to become a little more applied*.

Hence, the researchers thought that the CSO’s role was important and that they had the chance to affect the research agenda as well as research outcomes. This perception was not shared by the CSOs who saw themselves as on the fringes of the project with little possibility to influence either agenda or research. This feeling by the CSOs is illustrated in the following interview extracts with the CSO representative:

**Interviewee:** *To be clear, I think the intentions on paper were good. I think in my experience, they were special in our country. But from the beginning, in my opinion the planning of the project was further ahead than the good possibility to give a serious position to the patient users*.

***Interviewer*:** *So when you say it was further ahead, how were you co-opted into the project? What was your expected role in the beginning*?

***Interviewee*:** *I remember the first presentation I gave in this project, and that was the presentation I made based on the expectations that were given to me which I developed. And I was in the, I thought I was told there is a moment where I could present the expectations, the wishes, the needs and the problems of my patient group on behalf of what brain interfaced computers could give. And I made a presentation from my experience, some literature kind of thing where I am looking in the future where it could be very nice to develop things like … I had some examples. With Epilepsy, very important that there could be a possibility to alert them that between now and ten minutes there could be a seizure*.

***Interviewee*:** *So how did you find yourself getting to present in this project…were you invited*?

***Interviewee*:** *Yes, I was invited. I think the project was in a competition with other projects to get financed. And one of the good things of the project is that they had a description in their project for the role for users. They put it right on paper that the project was beginning and then someone thought oh yes we missed the users. And in a very late moment I was invited. I don’t know who found me but they thought about the project and I was very enthusiastic and I was invited*.

***Interviewer*:** *So did you think it was an afterthought, to invite if I can say a civil society organisation. By the way do your consider yourself a civil society organisation*?

***Interviewee*:** *Yes*.

There was a separate budget for the CSO involvement. This represented a small percentage of the overall project budget which raised the question of the importance of CSO input to the CSO representative. The CSOs had a sense that researchers were looking at them as volunteers who did not expect payment, a view with which the CSOs disagreed.

This project can probably best be described as an example of rather functional CSO involvement that did not have a transformative impact.

Respondents differed in their understanding of the term CSO as the following interview with the project coordinator demonstrates:

***Interviewee*:** *Yah. Well I think civil society at least for the dissemination part, people who are interested in brain research and brain computer interface and that can be students who are interested, can be primary school students, but can also be patients who would like to know whether they can use technology for their benefit, its people in academia, who may not be in the field but are actually interested in the findings but also the layman in the street who thinks brain computer interface is cool*.

***Interviewee*:** *Ok. And which sort of CSO have you got involved in the project*?

***Interviewee*:** *Mainly patient organisations*.

The understanding of the concept of CSO was somewhat different with the CSO representative involved in Case 2 who said the following:

***Interviewer*:** *How would you describe being a civil society organisation*?

***Interviewee*:** *For me it is a non-governmental, it is a self-help organisation that is ruled by citizens. Or how do you call it, citizens who are forming together a group trying to benefit their position. So I see a patient organisation as a civil society organisation*.

The difference in understanding the concept between the Scientific Coordinator is interesting, in that the scientific coordinator seems a bit unsure of the terminology while the CSO representative is much more confident. It is particularly interesting in this case especially when one considers the fact that the coordinators seemed to have had their hand forced by government and therefore by the need to have CSO involvement in their research before they could recognise the need or importance of CSO in research. This perhaps also reinforces why CSOs are often absent in research, because the different entities do not always speak or mix together and therefore have little awareness of each other.

#### 4.2.3 Case 3: Brain computer interfaces

The specific interest of the project was to link BCI technologies with other novel and emerging types of ICTs. Two specific ones that were mentioned as making the use of BCI technologies more suitable to users were virtual environments and affective computing, i.e. technologies that detect and react to emotional states of users. The specific purpose of the project was to develop technologies that would support severely disabled users. Such users with severe brain or spinal cord injuries lose significant aspects of their autonomy and the project's intended aim was to provide them tools to regain parts of this autonomy.

The overall aim of the project, was to develop technologies that would become available to disabled end users and that would also be commercially viable in the long term. The consortium consisted of seven partners in total with two CSO partners. CSO1 was an organisation representing disabled people while CSO2 was a hospital. The inclusion of CSOs in the project was for the purposes of establishing links between the researchers and end-users. The transformativity aspects are listed in Table 5:

**Table 5:** Summary of case 3.

	Researcher	CSO	Funder / Industry
**Expectations**	development of BCI technologies and achieving further scientific excellence publish in high quality outlets	Test the device on their constituents, i.e. individuals with severe brain or spinal cord injury	Focuses on the benefit for the eventual user of the technology expected impact underlines the economic aspect of the development
**Technology**	Expected to develop a prototype, not a product	Technology could not be tested due to lack of portability CSO1 remained unconvinced of practical applicability	Agnostic on actual technology; focus on benefit for users and economic benefits
**Assessment**	Success of project and technology development to be measured by peer reviewed publications	Test of actual technology in real-life conditions (did not happen)	General project review mechanisms; based on external expert (scientific) review
**Role**	Coordinator, main driver; the coordinator was not the leading scientist	User testing	Companies aimed to promote product development

One of the main reasons for including the CSOs was to have appropriate end user representation in the project. This was seen as a precondition to gain funding as expressed by CSO1: "I think they use end users to get funding. It’s more likely that if you’ve got end users involved then you’d get funding definitely." Interviewee CSO1 made it clear that practical relevance was sought for the project and the inclusion of end users was driven by this.

There is a limited number of CSOs with the specific expertise required here. The coordinator therefore reached out to CSO2, the not-for profit hospital, as they had experience of collaboration. One problem of the collaboration was the CSOs expectation that the device they were going to test was going to be portable, so that they could visit users in their homes to test it. It turned out that the technology was not at a portable stage yet. As a result it had to be installed in a lab. This made testing much more demanding, as patients had to be brought to the lab to use it, which, given the nature of their disabilities, was complex.

One area where conflicting expectations were particularly visible were follow-up projects. This project had a direct continuation project that arose from the work undertaken here. The new project did not include either of the two CSOs, however, but included a new CSO working in a similar area. This was an area of contention. Both CSOs knew of the follow-on project and were disappointed not to be included, but neither knew why they were not included. The coordinator's reason for choosing different CSOs was that the consortium should look different, otherwise he feared the project might not be funded.

Despite these various difficulties, however, the CSOs played an important role in the project. They were included early and had input in the development of the proposal and thus influenced the perception of the technology and potential output. The project can therefore be classified as demonstrating significant influence of the CSO as the following interview quote suggests:

***Interviewer*:** *Why do you think CSOs are a valuable partner for research or do you actually think they are a valuable partner for research*?

***Interviewee*:** *Yeah I do, I mean I think they are valuable, because normally I think they have actual experience of working very closely with the client groups. I think people in the Universities they don’t, they’ve not met a disabled person, a lot of them that I met on this project. But I work with them on this project so we actually practically tell them… give them that perspective. So yes I think it’s really important*.

With regards to the concept of CSOs, the concept of CSO may be specific to different languages and therefore difficult to translate as this interview quote suggests:

***Interviewer*:** *Okay. So, we are particularly interested in civil society organisations. Does that term mean anything to you*?

***Interviewee*:** *Not really, since it’s in a foreign language. I need to think what it could mean*.

Additionally, the question arises as to how to define a CSO when its initial focus shifts as the case was with CSO1 when it shifted focus to a business model. Suffice it to say, this did not sit well with the CSO representative in the project.

***Interviewee*:** *I would have liked the focus to continue on helping the most disabled people in society, directly as a donation based kind of charity service*?

This throws a whole new light on how CSOs ought to be looked at and what they ought to represent: should they be commercial entities and therefore for profit but still with some focus on helping special needs groups or should they not be considered as CSOs anymore? Reasons for a change of focus vary, but two possible reasons given were a) recession and b) managers of the CSOs believing a business model was far better than a charity oriented service.

#### 4.2.4 Case 4: Affective computing for augmented communication

The project used BCI to increase human capabilities and develop applications addressed primarily at patients, mainly children, suffering from a degenerative disease. The outcomes focussed on the augmentation of capabilities related to communication, learning, social participation and control of devices. During an interview the Coordinator of the project gave insight into why they embarked on an affective computing BCI project captured in the following interview segment:

So as we were working in assistive products in the 7th framework programme [EU funded], mainly all policies of inclusive work based BCI at first and BNCI developments in the latest course. So we were looking for our research problem that could benefit the users for the application of BNCI. Finally we find out…we realised the importance that communication problem has for some profiles of children with cerebral palsy. We felt that this could be one of the population that could benefit the most for improving the communication channels. These children are when they start development, intellectually normally 80% of cases more or less. But due to a lack of communication problem with the others within the environment, they become quickly delayed in their intellectual development through their childhood and arriving up to the adulthood. Therefore improving their communication could improve also their life expectancy and also their quality of life. And this is the main rationale of the project

The developed system was expected to be composed of four independent modules based on the latest advancements in BCI. These included signal processing, affective computing, augmented communication and bio-signal monitoring. The project successfully used computers to detect different emotive states and mind-sets of users through differences detected in voice intonation, and separating involuntary movements of patients from voluntary movements with the use of algorithms. The aim was to use these breakthroughs further to make, use and study prototypes to obtain information until the final product was reached. Out of the two CSOs involved, one was a non-profit, public utility founded by family and patients with the relevant national department and ministry, while the other was a research and clinical foundation and hospital specialising in rehabilitation of patients of various neuro degenerative diseases. The aspects of relevant to transformativity of CSO involvement are listed in Table 6:

**Table 6:** Summary of case 4.

	Researcher	CSO	Funder/Industry
**Expectations**	Actual technology product for sufferers of a degenerative disease in in order to improve their communication and social skills	A better integration of sufferers in everyday environments such as schools due to the intervention of the technology. Additionally, a desire not to put too much reliance on the technology but on understanding actual patient needs and problems	Funder: High importance placed on end-user participation particularly on patients and professionals like physiotherapists and rehabilitators who might potentially use the end product Industry: aimed at having better portfolio for companies they work for
**Technology**	Development of physical technological product	Emphasis not on the actual end product but on product capabilities in alleviating sufferers inability to communicate and be socially adept	Industry: Development of better sensors as a result of the project
**Assessment**	Testing of the product on end-users as a way of seeing its viability	Feedback on product after end-user tests Dissemination	Commercial viability of product
**Role**	Research and production of technology	Proposal writing End-user testing and dissemination WPs	Funders pushed for end-user participation Industry facilitates transition of the prototypes to commercial products

In this case, end-users and care professionals were involved in R&D tasks of the project from design to validation through the involvement of two CSOs who contributed since the very early stages of proposal writing and were given specific WPs relating to end-user testing and dissemination. Specifically, the CSO involvement in the project has been threefold: first, they were involved in defining the research problem, i.e. defining the user case studies and their relevance; two, they were involved in identifying the user group that might benefit from this research; three, they played a pivotal role in the dissemination of research results. They defined the research question according to the World Health Organisation (WHO) guidelines and identified the relevant user group. One of the CSOs was instrumental in changing scientists’ criteria of measuring the project’s success by replacing traditional mechanism of evaluation based on cognitive abilities, intelligence, ability to move and conventional psychometrics with enhancement in user performance, that is, the users’ device-aided actual output. This effectively changed the direction of the project. They also suggested generating new data on bio-signals for patients with degenerative diseases which was not available so far, and facilitated access to patients for this purpose. Further, they not only made a financial commitment from their own budget to participate in research as per the organisation’s constitution and goal, but also applied a novel idea of integrating patient testing in the daily culture of the organisation to work around the budget constraints. The involvement suggests that the CSOs had a great impact on the research outcomes of the project.

In defining the term or concept of CSO, the coordinator was not particularly sure of the specific term but gave an understanding of what CSOs were supposed to do:

***Interviewer*:** *So I was asking whether you know the term civil society organisation*.

***Interviewee*:** *I don’t know… I have never heard that before but I think I can understand what it means*.

***Interviewer*:** *Okay, what do you think it would mean*?

***Interviewee*:** *It’s a kind of…well from my point of view; it has a definition close to non-governmental organisation. So it is an organisation that is related to provide services to the society*.

In the case of CSOs themselves, defining what a CSO is, CSO1 defined civil society as an organisation that "thinks more about the social good than the financial gain". For him, the two are not polar opposites but a civil society organisation would think more about the one than the other. According to CSO1, the legal nature of foundations is confusing because some of them could be answerable to the general public and hence be genuine CSOs while others may have been set up either by the governments or companies to avoid social accountability and controls and could not be the real CSOs. For this reason, he is cautious in calling them a CSO and mentions this as a crucial factor. Here, it is notable that CSO2 is a privately owned, but government founded and publicly funded, non-profit organisation and there appears to be a conflict of interests among these two CSOs, also visible elsewhere during the interviews.

*There are foundations that come from social organisations and they are fantastic, I’m not saying that the others are not but underneath they can be companies or the government camouflaged*.

CSO2, a hospital, does not necessarily fall within the strict definition of a CSO but has been considered a CSO in this project because a) they are not-for-profit and b) they are a hospital i.e. an end-user/patient organisation. CSO2 interviewee hopes that they would be considered a CSO despite their organisational and funding mechanisms because after all, they represent the end-users in all aspects.

I am talking about in a more broad sense so our association represents users in all aspects, from medical to societal actual, so if you consider [us a] CSO…

In sum, the actors have agreed that they do not have a clear understanding of what a civil society organisation is but that the definition needs to be flexible and broad to include organisations that are active in the field of social good without a focus on financial benefits.

#### 4.2.5 Case 5: BCI for home monitoring

The aim of the project was to assist patients in their home setting with the aid of tele-monitoring technologies. The project aimed to develop BCI technologies that can assist severely disabled people in their home environments. The project aim is summed up in the following interview extract with the Coordinator:

We learnt from [previous project] that for people that have suffered some kind of disability, there is a very important moment, which is the moment when this change comes. When somebody for example has suffered an accident for example or brain damage or a spinal cord injury, they go to hospital and they have some kind of intervention and they stay there for a while doing some rehabilitation. But there is a moment when those people are discharged and they are brought *** home, that’s why the project is called “***”. Well we proposed in the project to provide means with technology for being able to go on with the relationship between clinicians and therapists with those patients at home. So apart from home support we are also providing tools for tele-monitoring the patients.

In particular, the technologies were intended to improve end-users autonomy and promote their independence. The intention was for end-users to have the freedom to carry on their lives as freely and as independently as possible so that their quality of life is improved. The tele-monitoring element is for the purpose of ensuring that there is a continuation of care when patients leave hospital. Such continuation of care enables clinicians and patients to communicate with each other and to allow any intervention in care much more easily should it be needed. This is summarised well in the following quote:

*And this is, for me, the most important thing of [Case 5], that we really get a step forward in bringing BCI to the end-user’s home and, so that’s one point, the other point is to integrate all the knowledge if you wish, that we have right now and that’s quite a bit as compared to like fifteen years ago when I started in that area. To integrate all that knowledge into one system, meaning that we will have a BCI with which you can communicate, with which you can control your environment, with which you can just have fun if you wish for entertainment. You can paint paintings or yeah, or, select music, or maybe download a video, browse the internet. And you can actually switch from one application to the other using in the end, hopefully, different brain signals. So, that’s, I would say, the main idea of [Case 5]*.

The key aspects of the case are summarised in Table 7:

**Table 7:** Summary of case 5.

	Researcher	CSO	Funder
**Expectations**	Make inroads in BCI research in order to alleviate patient suffering where patients have no autonomy to carry out everyday activities independently. Cultivation of new topics in order to advance the field of BCI research. Scientific publications	A realisation of positive results for disabled people. Particular interest in advancing research in the field of BCI	To advance research excellence in the field of BCI
**Technology**	Development of a tele-monitoring home system	Technology to advance well-being of patients	Industry: Commercial viability of the technology
**Assessment**	Viability of technology through feedback from users	End user testing and feedback	
**Role**	Coordination Lead scientist	Intermediary and link to interest patient groups and provide patient access to the researchers and technology developers for testing and feedback purposes. Linking industry to the end-users	Bringing a commercial angle as a way of making the product commercial read

The CSO in this case was only contributing to the work packages related to user training and project evaluation and related tasks and deliverables led by other consortium partners. They were not assigned any specific WPs because it was seen by researchers as an “administrative overload” on them. But their contribution in some specific research activities was visible and notable. For example, they provided patient access for testing purposes, provided feedback to researchers and technology developers about what works best for end-users and most importantly, worked as a link between industry and the end-users by helping define inclusion and exclusion criteria for prototypes and devices. It can be concluded that for this case, the CSO had limited influence.

When it came to the concept of CSO, there appears to be some confusion as to what a CSO might be, let alone what the term means. For example, initially, the scientific representative did not have a view of what a CSO might be. However, after giving it some thought, the interviewee came up with the following definition which was that a CSO is like a foundation or association whose aims are to:

….bring together ideas, concepts, results of different aspects of let’s say for example science but also from politics, bring them together like an ethics committee maybe and investigate, maybe investigate what are the consequences or the essence of different aspects, different results for the society

Interestingly, the CSO representative did not recognise the term CSO either and had the following to say:

*It’s not a familiar term but I understand what it means. It’s not a term that we would use in common language here, or in the UK. Um, no. Yeah, not familiar with it at all*.

It would appear that although there is an understanding of what the term CSO ought to stand for, do or achieve, the term itself may be rather meaningless to a few. Also, perhaps it is not what is in a term that is important but what the entities stand for and aim to achieve. This is because as the CSO representative seems to imply, it is a term that does not enjoy universal application.

## 5 Cross-case analysis and findings

Having given an introduction to the individual projects, we can now explore whether there has been evidence of transformativity and, if so, whether it is possible to identify factors or components that promote or inhibit transformation. In order to answer this question we start by discussing the components of transformativity to assess to which degree the different projects were transformed by CSO involvement. We then explore some factors or components that emerged as being relevant across the five cases and had an influence on transformativity.

### 5.1 Transformativity

In order to discuss the level of transformativity, we explore the expectations of participants, the perceptions of technology, assessment of the project, as well as the CSO’s role. To understand the expectations we begin by looking at the expectations of the researchers and then contrast these with those of the CSO representatives. We focus on the mutual expectations of these two groups and their expectations about the project and collaboration.

Researchers in Case 2 (computers and the brain) saw the project as a strong research project that would generate new theoretical knowledge of brain functions and their link to computers. They hoped to develop technologies to help patients, and move towards commercially available products. They had a clear and functional view of CSOs and their role. They saw inclusion of CSOs as a way of increasing the likelihood of winning funding, and associated them with specific tasks.

The CSO’s views of Case 2 mirrored the researchers’ to some degree, but they had a different focus. They expected concrete outcomes for their constituents and the allocation of funding for solving concrete problems, which did not materialise. They were also less clear how the research could actually bring the promised benefit for patients.

The mutual expectations between researchers and CSOs were clearer in case 1 (ICT support for disabled children). In this case the researchers were more aware of CSOs from the inception of the project, largely because the project was driven by one particular CSO. The researchers were aware of a mismatch between the requirement to develop a viable solution and the academic structure of stable projects that have to serve other purposes, such as training PhD students and delivering publications. The leading CSO was aware of this potential tension and saw their role as continuously reminding the researchers of the shared goal of creating a product.

The same pattern of expectations and tensions can be observed in the other three projects as well. Incentive structures for researchers were different from those of CSOs. The CSOs in each of our cases were keen to provide tangible results to their constituents, who are in all cases patients or people suffering from illness or medical conditions. The researchers were generally aware of this, but they had to balance this desire for practical solutions with a need to satisfy their institutional requirements. Notably this included the creation of publications, and other research-related metrics. Moreover, research funding rarely extends to the point where the product is commercially viable, leaving a fundamental gap between user expectations and the capability of research projects.

The influence of CSOs on the conception of the technology is another key component of transformativity. In case 2 this conceptualisation was driven by the CSO who had a clear view of what they wanted, namely a BCI-controlled orthosis to support weak upper limbs. The researchers understood this and worked towards it. This vision of the technology and how it could meet users’ needs was less clearly developed in the other projects.

The way in which the projects were evaluated was similar across the different projects, despite their different funders, sizes and approaches. All projects had an explicit evaluation process which periodically requested a number of metrics, sometimes linked with external evaluation by peer scientists and usually supported by a progress narrative. The mostly quantitative indicators used in evaluation included traditional scientific ones like numbers of publications and citations, but also aspects of engagement with society, such as number of contacts with patient groups or public engagement events. This component seemed to have a limited impact on the way in which CSOs influenced the project.

The final dimension to be discussed in this section is that of the role of the CSOs. In all cases they were official project partners, but their overall role differed greatly. In Case 1 the CSO set up a foundation with the explicit aim of creating the proposal and enabling the project. They successfully gathered money, including money from other CSOs which was a precondition of funding. They had a role in the evaluation of the project. However, most importantly, the president of the CSO kept informally in close contact with the researchers through frequent calls and meetings.

In Case 2 the CSO did not have a specific role. They did not have responsibility for work packages or deliverables to which CSOs were invited to contribute. Even work on public engagement was undertaken mostly by the researchers. In the other three projects the CSOs had specific tasks with regards to linking the project with end users, in all cases groups of patients or disabled individuals. However, these tasks took various shapes.

In Case 3 the CSO led the work package on user testing with full responsibility for related deliverables including the development of learning materials and organisation of stakeholder meetings.

In case 4 the CSO had been part of the consortium from the inception of the project, had thus contributed to the development of the proposal. They thus had three main roles: first, they were involved in defining the research problem, i.e. defining the user case studies and their relevance; second, they were involved in identifying the user group that might benefit from this research. Finally, they played a pivotal role in the dissemination of research results.

In Case 5 the CSO was relatively removed and called upon by researchers to provide contacts where required.

This brief comparison of the cases shows that there is a range of ways in which CSO involvement can transform research. The case where this transformative influence is most visible is Case 1 where the CSO substantially contributed to the funding, set out the vision, prepared the proposal and selected the partners. Case 2 is probably on the opposite end of the spectrum of the cases presented here in that the CSO, albeit part of the consortium, had no power to influence the research, its agenda, its execution or evaluation. Cases 3, 4 and 5 are located somewhere between the two extremes. The interesting question for this paper is whether these cases provide insight into which aspects or factors promote a transformative impact of CSOs.

### 5.2 Factors affecting transformativity

In this section we analyse which aspects of the project contributed, both positively and negatively, to a transformative influence of the CSOs on the research.

#### 5.2.1 Context of the project and funding requirements

The five projects were similar in many ways beyond their subject focus on BCI technologies. While two were nationally funded and three were EU projects, they all shared a strong (at least rhetorical) focus on the provision of solutions for end users, in most cases patients or disabled persons. They were not subject to external societal controversy. Case 1 was funded under a research programme that required significant external co-funding which explains the need for the CSO to raise funds. Case 2 followed a call that explicitly required CSO integration and broader societal valorisation of the project. All three EU projects worked under the assumption that end user involvement was intrinsically beneficial but also an expectation by the funder that would be taken into consideration by the evaluators.

These general aspects of project context are highly similar and therefore cannot account for the significant difference in the way in which the CSOs transformed the projects. A deeper look at other aspects of the projects is therefore required to provide an explanation for the varying levels of transformativity.

#### 5.2.2 History of collaboration and selection mechanisms

In Case 2 there was no history of collaboration between most of the partners. The consortium was assembled in response to a call. CSOs were approached on the basis of their subject focus but they did not know the researchers. Case 1 followed a completely different model in that the CSO was the driving force behind the proposal. The CSO not only co-funded the project but also created an organisation with the aim of writing proposals. The research partners were approached by the CSO. It is important to mention that the CSO had a history in attracting funding on the basis of research and had previously been involved in the successful development of drugs.

In Case 3 one of the CSOs involved was based in close physical proximity to the research coordinator and the two organisations had worked together successfully in the past. The second CSO in this case was new to the consortium without prior interaction. Case 4 was similar to this in that there was a long history of collaboration and close physical proximity between the coordinator and one CSO. In this case the second CSO also had prior experience of collaboration with the Coordinator. Moreover, all of the technical / scientific partners in this consortium had collaborated extensively in the past and very close relationship. Case 5, finally, was to a large extent a continuation of Case 4. However, while the technical / scientific membership of the consortium remained constant, the CSO members were changed, partly to reflect the changing application area of the BCIs and partly to ensure that the proposal did not look too similar to the previous project. The new CSO members were selected on the basis of their role in end-user evaluation and testing of the technology.

#### 5.2.3 Barriers and challenges of CSO collaboration

It is important to point out that the collaboration between CSOs and other partners, in particular researchers, raised specific challenges in all projects, even in the ones that worked well. In Case1 it was pointed out that the temporal aspect of research differed, in that the CSOs were pushing for quick results, which clashed with the researchers’ timetable, which for example, required a longer period for PhD research to be completed. This was one key component of the larger problem of incentive structures which differ significantly between CSOs and researchers.

Another key part of the incentive structure that emerged from all of the projects was the role of publications. Researchers are typically measured on the quantity and quality of their publications and are therefore keen to undertake research in a way that leads to high impact publications. This is of much less interest to CSOs who are interested in practical results and marketable products that can benefit their constituents.

These differences in incentives and expectations can be described as aspects of different organisational cultures that led to a number of other concerns. In Case 3 the researchers pointed to a different work ethic between different organisations with researchers in this particular project routinely working late into the night, if a problem required it, which they did not see reflected in other organisations. Another problem related to the background of the CSOs was lack of experience, in particular in terms of research they were supposed to undertake. CSO members in several of the cases were not experienced in the details of the methodology they were supposed to employ, which led to delays in the research. One particular issue that arose in Case 3 and Case 4 was that of research ethics. Given that the BCI work in these projects focused on vulnerable populations, notably disabled and ill users, working with them required ethics permissions. Gaining such permission can be a drawn-out complex process which was difficult for CSOs to engage with.

A further cultural issue was that of language. In one of the EU-funded projects one of the CSO partners did not have the capacity to communicate in English, which slowed down communication processes. But even in the nationally funded project the main project language was English, in which not all partners were fluent.

In addition there were numerous organisational and bureaucratic obstacles. One of these had to do with legal requirements for funding that impose certain constraints on which type of CSO is eligible. All of the CSOs in our cases had the legal status that allowed them to receive funding, but in some cases there had been earlier attempts to include other CSOs which failed due to their legal status. One frequently recurring issue was caused by bureaucratic requirements, e.g. the calculation of costs and the organisation of time keeping, which funders imposed on CSOs but for which they lacked the organisational capacities. Funding rules that allocated particular levels of funding to particular activities, sometimes requiring partners to operate at a financial loss raised problems as well.

A final barrier worth mentioning turned out to be the technology itself. There is a long history of research on BCIs, but at this point they are still relatively unreliable. Users’ ability to benefit from them is difficult to predict and far from uniform. This created problems for the CSOs who wanted to offer a service to their users who in several cases ended up disappointed because the technology did not live up to its promises.

#### 5.2.4 Enablers of CSO collaboration

Despite these manifold barriers, it was also possible to identify enablers, which in many cases mirror the barriers. One key component of successful CSO integration was a shared vision of the project and its likely outcomes. Where CSOs and other partners shared this vision they tended to find ways of overcoming barriers.

We could identify a number of components that contributed to the development of the shared vision of the project. The key to this shared vision seemed to lie in the relationship between the partners. Where the CSOs were treated as equal partners and were part of the development of the research agenda and the proposal, they generally reported that their view of the project corresponded with that of the researchers. This, in turn was helped by research experience of the CSO in general and by a history of successful collaboration between partners. Where such successful prior collaboration was present this contributed to the development of trust. Trust was also strengthened by frequent and close communication, often informal rather than formal communication. A further enabler of the development of these enablers was a structural similarity between partners, for example in terms of activities and organisational structures and organisational cultures. Geographical proximity could also support the development of the rapport between partners and contributed to several of these factors.

**Fig 2:**
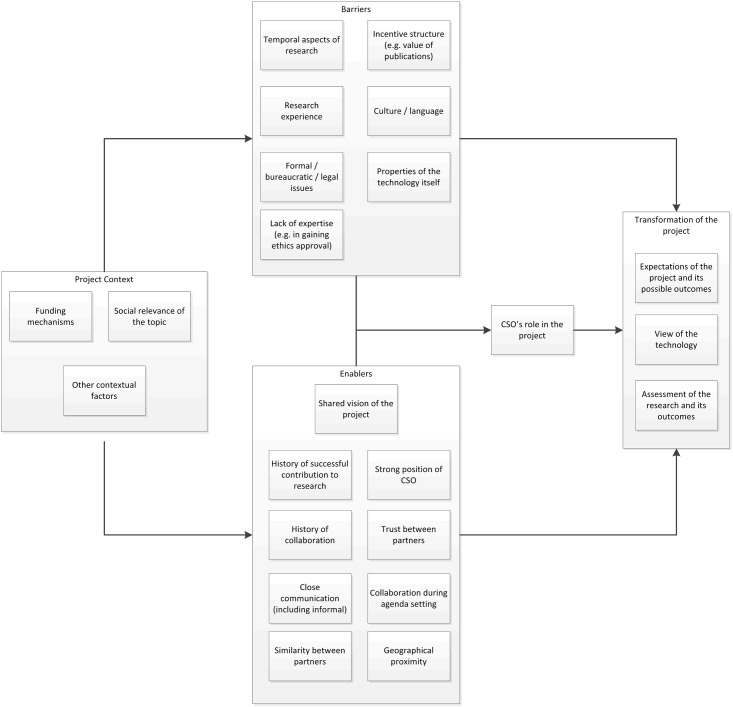
Graphical representation of the factors influencing the level of transformativity of CSO inclusion.

The discussion in this section shows that there is a complex mix of factors that can have an influence on the way in which CSOs can transform research projects. The following Fig 2 is an attempt to summarise and graphically represent the discussion in this section:

This figure can be read as follows: The degree to which the CSO inclusion has a transformative impact can be determined from changes to expectations, view of the technology and assessment of research outcomes. Our cross-case analysis has demonstrated that there are a number of barriers and enablers that can affect this transformation. In many cases these are directly related to the transformation, e.g. in that mutual expectations of partners in the project affect the expectations of the outcomes of the project. Both barriers and enablers are shaped by the external context, e.g. the funding structure or the topic area. A key factor that figures prominently in the discussion is the role of the CSO in the project. This role is shaped by barriers and enablers and it is a strong predictor of the eventual transformation. It is nevertheless important to underline that none of these relationships are simple or linear. This brings us to the final section where we discuss which conclusions can be drawn from this study.

## 6 Discussion and conclusion

In this final section we start by revisiting the concept of transformativity. This leads to a discussion of lessons learned and possible recommendations and possible further studies.

### 6.1 The concept of CSO

We started with the definition of a CSO proposed by the European Commission but, assuming that this definition might not be widely shared, we asked respondents how they defined the term. The finding was that the majority of our respondents, including some who worked for what we assumed to be CSOs were not familiar with the term. However, despite this initial lack of familiarity, the use of the term did not pose significant problems for our research. Respondents were generally able to relate to it quickly and guess its meaning. The important aspect of CSOs for this study, namely that they represent interests of that part of society which is to benefit from the research, was generally clear to respondents and they were mostly engaged in the various projects for this purpose.

One reason why this term turned out to be relatively unproblematic for our study was the subject area. In all cases presented here the CSOs played a part in research projects on BCI for ill, disabled and disadvantaged groups. The CSOs represented the interests of these groups. This does not mean that there were no grey zones. For example, one of the organisations we talked to was a private charitable hospital and it was not obvious whether this organisation should be considered a CSO.

It stands to reason that a tighter definition of CSOs will be required in future. Where, for example, research funding is dependent on the inclusion of CSOs, it needs to be clear which organisations would fulfil the criteria. Such a clearer definition will be required for legal and funding purposes but it is likely that this may backfire. We already saw in the earlier discussion that the EU’s definition of CSOs excludes some organisations that may well be in a position to fulfil the purpose of CSOs, namely to represent relevant societal stakeholders. Examples might be social enterprises. At the same time there will be organisations that fulfil the legal definition but pursue problematic special interests and whose inclusion would not promote the policy goals of public engagement.

The concept and definition of CSO will therefore require nuanced attention by policymakers and research funders to ensure that their inclusion is compatible with the goals of research policy. Despite these uncertainties concerning the terminology, however, our research did manage to discover some important insights concerning the question whether CSOs can have a transformative impact on research.

### 6.2 The concept of transformativity

Earlier, in section 3.3, “Indicators of Transformative Impact”, we have argued that transformation of research could be achieved by changing the expectations of the project and its possible outcomes, by changing the view of the technology or by changing the assessment of the research. All three factors were hypothesized to be affected by the role of a CSO within a project. Following the cross-case analysis we can state that evidence of transformation in all three respects could be observed.

It is important to note that the concept of transformativity is not fully determined by these factors. In fact, it turned out that there are fundamentally different levels on which research can be transformed. On the one hand we could observe transformation of the project’s trajectory where the CSOs gave input that changed specific aspects of data collection or user feedback. This modified the original vision of the more science-driven projects. In addition to this we could observe a deeper transformation that not only affected the specifics of the research project, but the research culture in which the research was undertaken. This latter type of transformation affected not only the project, but the networks of partners and even the nature and culture of the partners themselves, both CSO and other partners.

These different instantiations of transformativity are important, given that the scope of transformation can be limited by the project environment. The European Framework context, for instance, has a low potential for very transformative research as detailed research trajectories, milestones, deliverable and timetables must be laid out in advance of work being undertaken. In this case, the trajectory of research is a contractual affair, hence transformativity becomes a disruptive force that could potentially have legal ramifications. At the very least, it could serve to defund the research in question. Transformativity in terms of changes to the project work therefore has to take place outside of the project itself, notably at the level of research planning and project development. However, the broader notion of transformativity allows the transformation of research and organisational cultures beyond the immediate project.

### 6.3 Recommendations

Having established that CSO inclusion in research can have a transformative impact on BCI research, we can now consider which recommendations follow from this. The guiding assumption behind this is that transformation of research through CSO involvement is desirable because it leads research in the direction of social relevance and user acceptability. In our research we have found evidence that this is the case but we need to point out that this is not unequivocal. CSO inclusion in research can be marginal in the sense that the CSOs have little or no influence on the research. Maybe even more importantly, however, we need to point out that transformation of research through CSOs can lead to resistance. Such inclusion can complicate research, it requires resources and it changes established research structures and processes. Some researchers welcome this and there are growing efforts by funders and research policy makers to support this, for example by honouring research impact. At the same time this can run counter to established interests for whom the evaluation criteria of the scientific community are the gold standard of research quality and who are uncomfortable engaging with non-scientific partners and their interests.

Assuming that transformation is desired and that CSO involvement is regarded as a way of promoting broader engagement, social relevance and acceptability of research, our work points in the direction of a number of activities that can help promote such transformative impact.

Specifically with regards to BCIs, it appears that the current technical capabilities of these technologies are a major inhibitor of CSO inclusion. This appears to be based on high expectations of the CSOs involved, all of whom represented patients or individuals suffering from serious conditions, some of whom were disappointed by the lack of practical applicability and usability of the technology. This points towards an important duty of the researchers to realistically explain the current capabilities of the technology as well as the realistic improvement that can arise from a particular project.

All of the BCI projects we looked at in this project were portrayed predominantly in terms of support for people with illnesses or disabilities. In some cases there were other envisaged uses, such as the use of BCIs in gaming, but these were never in the foreground of the project. As a consequence the projects were all seen by all participants as well as their social environment as beneficial and raised no broader concerns. It is important to note that with the further development of the BCI technology initial commercial products are coming to market which have non-therapeutic purposes including gaming and neuro-feedback. These may raise broader social concerns which could lead to a more difficult environment for CSOs and researchers to navigate.

The specific characteristics of BCIs form part of the broader matrix of relationships between different types of partners which are crucial to ensure that CSO participation can be transformative. Barriers are manifold and refer to the external context as much as the capabilities of different partners and the technology itself. The enablers, on the other hand predominantly point to the relationship between the partners and their mutual expectations. Recommendations to facilitate transformative CSO involvement can therefore on the one hand aim at removing or minimising the barriers. On the other hand they can point towards ways of improving relationships.

Recommendations for policymakers and funders therefore include activities to remove barriers. This includes fairly uncontroversial ones such as the reduction of bureaucracy and the facilitation of funding and participation models that put less of an onus on CSOs which often lack the organisational capacity to deal with them. It also includes raising awareness and creating an environment that is conducive to CSO inclusion, for example by celebrating positive outcomes or emphasising roles that CSOs often take, such as dissemination and outreach.

Some possible recommendations to funders and policymakers could be more contentious. If the research culture is to be changed to allow for greater emphasis on social outcomes, then this might mean a redefinition of scientific excellence. The traditional scientific focus on publishing high impact papers was clearly identified as an impediment to successful CSO inclusion. One way of changing this would be a broadening of scientific excellence to include societal impact. If the purpose of research is to address social challenges, then the success of research in this regard could be weighted more heavily in research evaluation.

In addition to these recommendations on a funding and policy level one can also make suggestions to researchers and CSOs. At the core of these recommendation is the aim to establish a good and trusting working environment between different types of partners. For researchers it is important to clarify why they seek CSO involvement and which role they have in mind for CSOs. If they intend to remain strongly in control of the research, it is worth saying so early. They should also be clear about the motivation to undertake the research in the first place and their abilities and capabilities. They should furthermore be realistic in terms of what a piece of research can achieve.

The recommendations to CSOs mirror those to researchers to a large degree. CSOs with an interest in research should be clear about what they expect and why they want to engage. They should highlight their abilities and how the research fits within their mission. As we have seen from the case studies, CSOs that can actually provide funding for research have a very strong position in guiding it. Such funding may be a good way of accessing research. In order to be able to cooperate with researchers, CSOs should also develop their research capabilities and experience.

Some of these recommendations are simply good project practice, such as being honest and open to one another, communicating well and often, and being willing to consider one’s position in the light of good arguments. Others will require time to come to fruition. Successful collaboration history was shown to be an important factor in enabling future collaboration. Such a history can only develop if the different kinds of partners are willing to engage, have the time and the institutional context where such collaboration flourishes. Some aspects require more fundamental change and modifications of the structure and incentives of the research system. Whether this will happen will be determined to a large degree by whether policymakers are willing to promote research as a response to social challenges, which is part of a greater debate around research policy.

### 6.4 Contribution to knowledge and further research

The discussion of the role of the public in research has been ongoing for some time. Strong arguments for public engagement have led to a significant policy push towards it. Research policy makers see a number of interlinking advantages that range from higher democratic legitimacy of research and its findings to instrumental improvements of the research itself. Despite this high level of interest there has been very little empirical research that shows the daily organisational reality of how such public engagement can be implemented. This paper contributes to filling this gap in knowledge by providing a detailed account of the way in which CSOs can be integrated into BCI research.

Our findings confirm some arguments that can be found in the literature and they also show that realities of project management and execution have an important role to play in facilitating collaboration. More importantly, we provide a collection of the barriers and enablers that influence the success of such collaboration. Our most important contribution, however, is that we show that CSO involvement can radically transform BCI research. It may not be very surprising to argue that user involvement improves research. What we believe is surprising is that CSO involvement can radically change the way in which research is perceived and executed. Such radical transformation is by no means a given, and is a rare phenomenon. However, we show that it is possible and that it can lead to results that benefit research users as well as the researchers themselves.

We believe that these findings have radical potential. If research policy is serious about desiring public engagement and promoting CSO involvement as one aspect of this, then it needs to consider the framework in which such collaboration can occur. Taking public engagement seriously can disrupt the current business as usual and lead to higher impact. The cost would be a willingness to move beyond current research models, as we outlined in the recommendations above.

These conclusions are exciting for policymakers and can feed into the development of policy. However, this paper primarily is aimed at a different audience, namely that of the scientific and technical experts who undertake the research. We chose to focus on BCIs for methodological reasons, as it allowed us to closely compare five cases that are located in the same field. BCIs are a technology that fulfils the criteria for public engagement by holding significant promises to improve the lives of users while simultaneously raising social and ethical concerns. The immediate audience of this paper is therefore BCI researchers who can learn that it may be worthwhile to deeply engage with civil society in their research. We also provide this audience with a set of recommendations on how to make such collaboration work. Our findings show that the characteristics of BCIs played an important role in determining the level to which this involvement was transformative. On the other hand the study also shows that many of the important factors were independent of the technology in question. Furthermore the CSOs in this study were structurally similar in that they all represented patients or people with disabilities or conditions who could be supported by the successful implementation of BCIs.

Further research would therefore have to look into other types of CSOs and how their role could affect research. It would also be interesting to look into other fields of research beyond BCIs to understand in more detail how the subject of research affects CSO integration. In this study we focused on the processes taking place within research and all data was collected from live projects. A further step would therefore be to look at the actual outcomes of the research to better understand how and to what degree tranformativity was achieved.

While much interesting research remains to be done, we believe that this study has shown that in the case of BCI research the inclusion of CSOs can have transformative impact. We have provided evidence of this impact as well as the factors that affect it. Researchers, CSOs and policymakers can all benefit from the lessons learned to ensure that research is brought closer to social concerns via the inclusion of CSOs.

Our study therefore makes an important contribution to the understanding of the practice of research involving CSOs. It provides a theoretical basis and empirical evidence concerning the question whether CSOs are transformative. It thereby contributes both to the immediate practice in BCI research and broader questions of research governance and research policy beyond BCIs.

## Supporting information

S1 Appendix Consolidated criteria for reporting qualitative studies. (DOCX)

S2 Appendix Coding tree. (DOCX)

S3 Appendix Interview questions. (DOCX)

S4 Appendix Case study report template. (DOCX)

S5 Appendix Data access. (DOCX)
