# Supplementary material for: Improving brain computer interface research through user involvement - The transformative potential of integrating civil society organisations in research projects
Source: PLoS One. 2017 Feb 16;12(2):e0171818. doi: 10.1371/journal.pone.0171818 (PMC5313172; doi:10.1371/journal.pone.0171818)
Supplement: S1 Appendix — (DOCX) [file pone.0171818.s001.docx]

**Appendix 1: Consolidated criteria for reporting qualitative studies (COREQ): 32-item checklist**

| No | Item | Guide questions/description | How the criteria were met by the research presented in this paper |
| --- | --- | --- | --- |
| Domain 1: Research team and reflexivity |  |  |  |
| Personal Characteristics |  |  |  |
| 1. | Interviewer/ facilitator | Which author/s conducted the interview or focus group? | Authors 1 and 2 were the coordinator / senior research fellow and conducted all interviews |
| 2. | Credentials | What were the researcher's credentials? *E.g. PhD, MD* | Both PhD |
| 3. | Occupation | What was their occupation at the time of the study? | Professor, Senior Research Fellow |
| 4. | Gender | Was the researcher male or female? | 1 male, 1 female |
| 5. | Experience and training | What experience or training did the researcher have? | Both experienced researchers |
| Relationship with participants |  |  |  |
| 6. | Relationship established | Was a relationship established prior to study commencement? | No |
| 7. | Participant knowledge of the interviewer | What did the participants know about the researcher? e*.g. personal goals, reasons for doing the research* | Participants were sent an information sheet about the project as a whole, which formed part of the ethics approval. No personal information was shared. |
| 8. | Interviewer characteristics | What characteristics were reported about the interviewer/facilitator? e.g. *Bias, assumptions, reasons and interests in the research topic* | None |
| Domain 2: study design |  |  |  |
| Theoretical framework |  |  |  |
| 9. | Methodological orientation and Theory | What methodological orientation was stated to underpin the study? *e.g. grounded theory, discourse analysis, ethnography, phenomenology, content analysis* | This was an interpretive case study. Data analysis was inspired by Grounded Theory but due to the complexity of the project the eventual data analysis was more structured, thus closer to thematic analysis. |
| Participant selection |  |  |  |
| 10. | Sampling | How were participants selected? *e.g. purposive, convenience, consecutive, snowball* | Purposive sampling on the basis of a prior survey |
| 11. | Method of approach | How were participants approached? e*.g. face-to-face, telephone, mail, email* | Initial contact was established per email. Most interviews were conducted by Skype / telephone. Some interviews were conducted face to face. |
| 12. | Sample size | How many participants were in the study? | Each case had at least three respondents, some more (see Table 2: Data on Case Study Projects) |
| 13. | Non-participation | How many people refused to participate or dropped out? Reasons? | This data was not collected. Anecdotally, there was very little non-participation. |
| Setting |  |  |  |
| 14. | Setting of data collection | Where was the data collected? e*.g. home, clinic, workplace* | The telephone interviews were mostly held from work places. The same applies to face-to-face interviews. One face-to-face interview took place in a public place, for convenience of meeting |
| 15. | Presence of non-participants | Was anyone else present besides the participants and researchers? | No |
| 16. | Description of sample | What are the important characteristics of the sample? *e.g. demographic data, date* | See Table 2: Data on Case Study Projects |
| Data collection |  |  |  |
| 17. | Interview guide | Were questions, prompts, guides provided by the authors? Was it pilot tested? | For Interview questions see appendix III below. The research approach was tested using a pilot case. |
| 18. | Repeat interviews | Were repeat interviews carried out? If yes, how many? | No. |
| 19. | Audio/visual recording | Did the research use audio or visual recording to collect the data? | Yes, all interviews were audio recorded. |
| 20. | Field notes | Were field notes made during and/or after the interview or focus group? | In some cases notes were taken that informed the analysis |
| 21. | Duration | What was the duration of the interviews or focus group? | Interviews lasted between 30 and 90 minutes |
| 22. | Data saturation | Was data saturation discussed? | Data saturation was reached for the overall project. |
| 23. | Transcripts returned | Were transcripts returned to participants for comment and/or correction? | No. We have not had good experience with this, as participants do not tend to comment on transcripts. |
| Domain 3: analysis and findings |  |  |  |
| Data analysis |  |  |  |
| 24. | Number of data coders | How many data coders coded the data? | Two individuals coded the five cases described here, the same who also did the data collection. |
| 25. | Description of the coding tree | Did authors provide a description of the coding tree? | See below, annex III. |
| 26. | Derivation of themes | Were themes identified in advance or derived from the data? | Main themes were agreed upon in advance. Sub-nodes emerged from the data and were agreed by the project partners. |
| 27. | Software | What software, if applicable, was used to manage the data? | NVivo server, version 10 |
| 28. | Participant checking | Did participants provide feedback on the findings? | No |
| Reporting |  |  |  |
| 29. | Quotations presented | Were participant quotations presented to illustrate the themes / findings? Was each quotation identified? e*.g. participant number* | In the full case description there are quotations that are linked to the individuals’ roles.  In this paper, due to an attempt to keep the paper as succinct as possible, the quotations were kept to a minimum. |
| 30. | Data and findings consistent | Was there consistency between the data presented and the findings? |  |
| 31. | Clarity of major themes | Were major themes clearly presented in the findings? |  |
| 32. | Clarity of minor themes | Is there a description of diverse cases or discussion of minor themes? |  |
