# Supplementary material for: Improving brain computer interface research through user involvement - The transformative potential of integrating civil society organisations in research projects
Source: PLoS One. 2017 Feb 16;12(2):e0171818. doi: 10.1371/journal.pone.0171818 (PMC5313172; doi:10.1371/journal.pone.0171818)
Supplement: S2 Appendix — (DOCX) [file pone.0171818.s002.docx]

**Appendix 2: Coding Tree**

| Name |  | Sources | References |
| --- | --- | --- | --- |
| _Gold dust quotes |  | 21 | 29 |
| Collective experiences |  | 15 | 20 |
| CSO |  | 188 | 1668 |
| Challenges faced by CSO |  | 40 | 119 |
| CSO influence on the project |  | 90 | 210 |
| CSO motivation to participate | 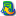 | 50 | 114 |
| Definition of CSO |  | 93 | 155 |
| Evaluation of CSO participation in project |  | 47 | 111 |
| Grounds of Selection |  | 68 | 129 |
| Nature and aim of CSO |  | 49 | 113 |
| Reasons for CSO involvement |  | 72 | 140 |
| Role of CSO in the project |  | 130 | 473 |
| Timing of CSO involvement |  | 60 | 103 |
| Expectations |  | 18 | 32 |
| Conflicting expectations |  | 58 | 122 |
| Expectation management |  | 35 | 58 |
| Expectations by CSOs |  | 83 | 225 |
| Expectations by funders |  | 30 | 56 |
| Expectations by industry |  | 34 | 87 |
| Expectations by researchers |  | 96 | 240 |
| Expectations by society |  | 23 | 37 |
| Publications from the project |  | 24 | 37 |
| Shared Expectations |  | 16 | 20 |
| Influence factors |  | 134 | 944 |
| barriers |  | 22 | 44 |
| enablers |  | 120 | 844 |
| Impact of funding on projects |  | 24 | 56 |
| Project |  | 233 | 2995 |
| Background |  | 64 | 125 |
| Budget |  | 33 | 51 |
| Challenges and problems |  | 81 | 233 |
| Consortium |  | 94 | 218 |
| Dissemination |  | 50 | 119 |
| Division of tasks |  | 82 | 227 |
| Duration of project |  | 10 | 11 |
| Ethics of the project |  | 43 | 93 |
| Evaluation of the project |  | 81 | 157 |
| Funding source and mechanisms |  | 30 | 46 |
| Good practice in project |  | 71 | 176 |
| Impact |  | 36 | 96 |
| Institutional embedding of CSO in projects |  | 45 | 115 |
| Lessons learned |  | 42 | 77 |
| Meetings |  | 48 | 80 |
| Openness of research process |  | 25 | 35 |
| Organizing the research process |  | 44 | 115 |
| Project management |  | 85 | 230 |
| Project management meetings |  | 48 | 76 |
| Project outcomes |  | 58 | 130 |
| Public interest |  | 64 | 187 |
| Purpose of the project |  | 61 | 114 |
| Beneficiaries |  | 88 | 181 |
| Project Innovation |  | 43 | 76 |
| Scientific or technological innovation |  | 36 | 76 |
| Social innovation |  | 27 | 42 |
| Social context |  | 41 | 133 |
| Communication with society |  | 26 | 44 |
| Role of industry |  | 14 | 19 |
| Role of media |  | 14 | 21 |
| Stakeholders |  | 48 | 86 |
| Topic of the project |  | 64 | 101 |
| Unplanned occurrences |  | 24 | 34 |
| Recommendations |  | 11 | 18 |
| Cooperation in general (recommendation) |  | 41 | 67 |
| Project-related recommendations |  | 44 | 74 |
| Recommendations for CSOs |  | 34 | 58 |
| Recommendations for funders |  | 53 | 89 |
| Recommendations for industry |  | 5 | 5 |
| Recommendations for policymakers |  | 47 | 82 |
| Recommendations for researchers |  | 48 | 93 |
| Relationship dynamics |  | 19 | 34 |
| Communication |  | 82 | 230 |
| Cooperation in general (description) | 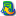 | 59 | 189 |
| Different cultures, researchers - CSOs |  | 58 | 122 |
| Mutual understanding, translations |  | 40 | 77 |
| Shared vision of the project |  | 18 | 24 |
| Uninvited interaction or relationship |  | 6 | 7 |
| Respondent |  | 6 | 11 |
| Emotional response |  | 51 | 116 |
| Experience of vulnerability |  | 16 | 28 |
| Power of Social Coordination |  | 11 | 20 |
| Respondent's background |  | 96 | 191 |
| Respondent's motivation |  | 68 | 152 |
| Respondent's organisation |  | 47 | 99 |
| Respondent's reason to participate |  | 16 | 20 |
| Respondent's role in the project |  | 64 | 163 |
| Rhetoric |  | 16 | 23 |
| Societal relevance of the project |  | 47 | 92 |
