# Supplementary material for: Improving brain computer interface research through user involvement - The transformative potential of integrating civil society organisations in research projects
Source: PLoS One. 2017 Feb 16;12(2):e0171818. doi: 10.1371/journal.pone.0171818 (PMC5313172; doi:10.1371/journal.pone.0171818)
Supplement: S3 Appendix — (DOCX) [file pone.0171818.s003.docx]

**Appendix 3: Interview Questions**

**Prior to the questions we need to ensure informed consent, so there needs to be a section on what we will do with the data and an assurance of anonymity. We need written records of informed consent.**

- **Are you happy for us to record this interview?**

**Description of the overall project**

- Could you very briefly describe the project?
- What is your personal role in the project?
- How did you become involved?
- Is there broader public interest in the project?
- How do you feel about the project?

**Meaning of the CSO participation for the overall project**

- How were the CSOs selected to be included in the project?
- Why is / are CSOs involved in the project?
- When (at what stage of the project) were they included?
- Which percentage of the budget / efforts are spent on / by CSOs?
- How do/did the CSOs influence the course of the project?
- What further effects did the participation have inside and outside the project?

**Definition of CSO**

- What do you think is a CSO?
- Why are CSOs a valuable partner for this research project and for research projects in general?

**Conflicts**

- Did you experience any conflicts in the project related to the participation processes?
- How did you solve them?

**Evaluation**

- How did/will you in the project evaluate the success of the project as a whole?
- How did/will you in the project evaluate the success of the participation process?
- Looking back at the participation process in your project, what do you think went well, what do you think went wrong?
- What consequences did the CSO participation have for the all involved organizations (CSOs, research partners others)?
- Do you think the other partners (CSOs, researchers, others) would regard the project as a success? Would they regard the participation as a success?
- Could you rate the cooperation between CBOs and science in your project on a scale from 1 to 10, whereas 1 is very bad and 10 is excellent?

**Interviewee-related questions**

- How did you get involved personally?
- What did you personally expect from the project?
- How would you evaluate the project from your personal perspective?

**Follow-on**

- Who else do you think has a view on CSO participation in this project? Who should we include in our analysis?
- What guidelines / recommendations could help your work?
- Can you point us to further documents (e.g. website, deliverables, publications) that we should be aware of in order to understand the project and the role of CSOs in it?
