# Supplementary material for: Improving brain computer interface research through user involvement - The transformative potential of integrating civil society organisations in research projects
Source: PLoS One. 2017 Feb 16;12(2):e0171818. doi: 10.1371/journal.pone.0171818 (PMC5313172; doi:10.1371/journal.pone.0171818)
Supplement: S4 Appendix — (DOCX) [file pone.0171818.s004.docx]

**Appendix 4: Case Study Report Template**

Table of contents

[***Background 1***](#_Toc369170484)

[***The CONSIDER Project 2***](#_Toc369170485)

[***CONSIDER Case Studies 2***](#_Toc369170486)

[***Short Description 3***](#_Toc369170487)

[***Abstract 3***](#_Toc369170488)

[**Keywords 3**](#_Toc369170489)

[***Table of contents 3***](#_Toc369170490)

[***The Project 6***](#_Toc369170491)

[**Project Topic / Theme 6**](#_Toc369170492)

[**Purpose of the project 6**](#_Toc369170493)

[**Project Context 6**](#_Toc369170494)

[**Project Facts 6**](#_Toc369170495)

[Consortium size and membership 6](#_Toc369170496)

[Funding source 6](#_Toc369170497)

[Budget 6](#_Toc369170498)

[Discipline 6](#_Toc369170499)

[Content 6](#_Toc369170500)

[Basic / applied research 6](#_Toc369170501)

[Beneficiaries 6](#_Toc369170502)

[Project management structure 6](#_Toc369170503)

[Dissemination and communication 6](#_Toc369170504)

[Conflict resolution 6](#_Toc369170505)

[Ethics 6](#_Toc369170506)

[Evaluation mechanisms 6](#_Toc369170507)

[What is specific about this project? 6](#_Toc369170508)

[**Expectations 6**](#_Toc369170509)

[Researcher expectations 6](#_Toc369170510)

[CSO expectations 7](#_Toc369170511)

[Funder expectations 7](#_Toc369170512)

[Other Expectations 7](#_Toc369170513)

[***Civil Society Organisation in the Project 7***](#_Toc369170514)

[**History of collaboration 7**](#_Toc369170515)

[**Grounds and mechanism of selection / inclusion 7**](#_Toc369170516)

[**Role of CSO, practice of participation 7**](#_Toc369170517)

[**Tasks within the project 7**](#_Toc369170518)

[**Other external influences 7**](#_Toc369170519)

[**Conflicts 7**](#_Toc369170520)

[**Evaluation of participation 7**](#_Toc369170521)

[***Research on the Case 7***](#_Toc369170522)

[**Reason for selection 7**](#_Toc369170523)

[**Data sources 7**](#_Toc369170524)

[**Data collection 7**](#_Toc369170525)

[**Data analysis 8**](#_Toc369170526)

[***Lessons Learned, Recommendations 8***](#_Toc369170527)

[**What worked particularly well according to the stakeholders? 8**](#_Toc369170528)

[**According to the stakeholders, what were notable problems? 8**](#_Toc369170529)

[**What are the lessons learned (from the point of view of the case under consideration)? 8**](#_Toc369170530)

[**Which recommendations do stakeholders make? 8**](#_Toc369170531)

[For policymakers 8](#_Toc369170532)

[For funders 8](#_Toc369170533)

[For researchers 8](#_Toc369170534)

[For CSOs 8](#_Toc369170535)

[For industry 8](#_Toc369170536)

[Project structure / management 8](#_Toc369170537)

[Other recommendations 8](#_Toc369170538)

[***Model of Participation 9***](#_Toc369170539)

[***Critical Reflection 10***](#_Toc369170540)

[**What recommendations do we think arise from the case? 10**](#_Toc369170541)

[**How does the case relate to literature / analytical grid? 10**](#_Toc369170542)

[***References 10***](#_Toc369170543)
