# Supplementary material for: Improving brain computer interface research through user involvement - The transformative potential of integrating civil society organisations in research projects
Source: PLoS One. 2017 Feb 16;12(2):e0171818. doi: 10.1371/journal.pone.0171818 (PMC5313172; doi:10.1371/journal.pone.0171818)
Supplement: S5 Appendix — (DOCX) [file pone.0171818.s005.docx]

**Appendix 5: Data Access**

The research presented in this paper was undertaken under the approval of the Faculty of Technology Human Research Ethics Committee of De Montfort University, approval number: 1314/197

The condition of approval was to ensure confidentiality of individual respondents. This is reflected in the participant information sheet and consent form.

Upon analysis of the data it became clear that individual interviews could not be anonymised to the point where individuals would not be recognisable. Moreover, even the aggregate case studies that are discussed in this paper are incapable of being anonymised. Individual cases are so specific that an observer familiar with the relatively small field of BCI research would be capable of identifying individuals from anonymised quotes. It was therefore decided not to make the data or the case analyses publicly available.

However, in order to ensure that independent experts and reviewers can assess the quality of the data analysis and the resulting narrative, data access will be made available on an individual basis. Individuals desiring access to data need to contact the Faculty of Technology Human Research Committee of De Montfort University (TRIOSupport@dmu.ac.uk). Upon signature of a confidentiality agreement that corresponds to the conditions of the ethics approval, individuals will be given access to the data.
